# Eukaryotic initiation factor 3a promotes the development of diffuse large B-cell lymphoma through regulating cell proliferation

**DOI:** 10.1186/s12885-024-12166-0

**Published:** 2024-04-08

**Authors:** Hongkun Sun, Juanjuan Shang, Xiao Liu, Shuai Ren, Shunfeng Hu, Xin Wang

**Affiliations:** 1 Department of Hematology, Shandong Provincial Hospital, Shandong University, 250021 Jinan, Shandong China; 2 Department of Hematology, Shandong Provincial Hospital Affiliated to Shandong First Medical University, No. 324, Jingwu Road, 250021 Jinan, Shandong China; 3 Department of Hematology, Binzhou Medical University Hospital, 256603 Binzhou, Shandong China; 4 Department of Oncology, Zibo Central Hospital, 255016 Zibo, Shandong China

**Keywords:** Diffuse large B-cell lymphoma, eIF3a, Cell proliferation, Cell apoptosis, Tumor immune, Chemosensitivity

## Abstract

**Background:**

One-third of diffuse large B-cell lymphoma (DLBCL) patients suffer relapse after standard treatment. Eukaryotic initiation factor 3a (eIF3a) is a key player in the initial stage of translation, which has been widely reported to be correlated with tumorigenesis and therapeutic response. This study aimed to explore the biological role of eIF3a, evaluate its prognostic and therapeutic potential in DLBCL.

**Methods:**

RNA-seq datasets from GEO database were utilized to detect the expression and prognostic role of eIF3a in DLBCL patients. Protein level of eIF3a was estimated by western blot and immunohistochemical. Next, DLBCL cells were transfected with lentiviral vector either eIF3a-knockdown or empty to assess the biological role of eIF3a. Then, samples were divided into 2 clusters based on eIF3a expression and differentially expressed genes (DEGs) were identified. Function enrichment and mutation analysis of DEGs were employed to detect potential biological roles. Moreover, we also applied pan-cancer and chemosensitivity analysis for deep exploration.

**Results:**

eIF3a expression was found to be higher in DLBCL than healthy controls, which was associated with worse prognosis. The expression of eIF3a protein was significantly increased in DLBCL cell lines compared with peripheral blood mononuclear cells (PBMCs) from healthy donors. eIF3a knockdown inhibited the proliferation of DLBCL cells and the expression of proliferation-related proteins and increase cell apoptosis rate. Besides, 114 DEGs were identified which had a close linkage to cell cycle and tumor immune. eIF3a and DEGs mutations were found to be correlated to chemosensitivity and vital signal pathways. Pan-cancer analysis demonstrated that high eIF3a expression was associated with worse prognosis in several tumors. Moreover, eIF3a expression was found to be related to chemosensitivity of several anti-tumor drugs in DLBCL, including Vincristine and Wee1 inhibitor.

**Conclusions:**

We firstly revealed the high expression and prognostic role of eIF3a in DLBCL, and eIF3a might promote the development of DLBCL through regulating cell proliferation and apoptosis. eIF3a expression was related to immune profile and chemosensitivity in DLBCL. These results suggest that eIF3a could serve as a potential prognostic biomarker and therapeutic target in DLBCL.

**Supplementary Information:**

The online version contains supplementary material available at 10.1186/s12885-024-12166-0.

## Introduction

Diffuse large B-cell lymphoma (DLBCL) is the most common subtype of lymphoma, which accounts for about 30–58% of NHL [1]. In the era of new drugs for tumor treatment, such as anthracyclines and monoclonal anti-CD20 antibody, the prognosis of DLBCL patients has been significantly improved [2, 3]. However, a recent large-scale survey has found the cure rate of DLBCL range from only 52.8–68.9% in the USA, and about one-third of DLBCL patients eventually become relapsed [4, 5]. Therefore, novel molecular targets and treatment strategies are urgently needed to improve the prognosis of DLBCL [6].

Translation initiation factors, which include at least 10 kinds in eukaryotes, play key roles in translation process [7]. Among them, eukaryotic initiation factor 3 (eIF3) participates in the cap-dependent translation initiation and depolymerization of 80 S ribosomes during the termination of translation process, which prevents the premature binding of 40 S subunit to 60 S subunit, and regulates the initial stage of translation [8, 9]. eIF3a, also known as p150 and eIF3-p170, is the largest subunit and functional core of eIF3, which was firstly purified from rabbit reticulocyte lysate and mainly distributed in the cytoplasm [10]. Recent investigations have implicated that eIF3a was upregulated in several solid tumors, such as urinary bladder cancer (UBC) and hepatocellular carcinoma (HCC) [11–13], eIF3a expression has been found to be associated with prognosis of tumor patients [12, 14]. However, the role of eIF3a in tumor development is controversial. Previous studies showed that the stable suppression of eIF3a could increase cell doubling time and change cell sensitivity to various cycle regulators [15], indicating the pro-oncogenic role of eIF3a in tumor development [12, 16, 17]. In contrast, other research found that eIF3a could protect low-grade malignancies from progressing to high-grade types [14, 18], suggesting the potential tumor-suppressive role of eIF3a. However, the expression level and biological role of eIF3a in hematological malignancies, especially in DLBCL have not been elucidated yet.

Our study aimed to evaluate the expression and biological function of eIF3a in DLBCL. We demonstrated that eIF3a was overexpressed in DLBCL compared with healthy control. DLBCL patients with high eIF3a expression displayed worse overall survival (OS). The expression of eIF3a protein was significantly increased in DLBCL cell lines compared with peripheral blood mononuclear cells (PBMCs) from healthy donors. eIF3a knockdown could inhibit the proliferation of DLBCL cells, and increase the cell apoptosis rate. In addition, function analysis revealed eIF3a had a close linkage to cell cycle and tumor immune. Mutation analysis found there were great discrepancy in several pathways and druggable categories between eIF3a-related differentially expressed genes (DEGs) mutation groups and non-mutation groups. Moreover, eIF3a expression was also found to be associated with chemosensitivity in DLBCL. These results indicate that eIF3a might be a promising prognostic biomarker and therapeutic target in DLBCL treatment.

## Materials and methods

### Data acquisition

The study datasets contained GSE25638 (*n* = 97, including 26 DLBCL and 6 B cell samples from healthy controls), GSE31312 (*n* = 498), GSE23501 (*n* = 69) and GSE181063 (*n* = 1037) from Gene Expression Omnibus (GEO) database (https://www.ncbi.nlm.nih.gov/geo/), and TCGA-DLBCL (*n* = 47) from The Cancer Genome Atlas (TCGA) database (https://www.cancer.gov/). Pan-cancer dataset from UCSC (https://xenabrowser.net/) and TCGA were utilized to explore the expression and prognostic value of eIF3a in other malignancies.

### Patient samples

This study was approved by the Biomedical Research Ethic Committee and the consent form was signed. Paraffin-embedded specimens were obtained from 44 newly diagnosed DLBCL patients and 32 reactive hyperplasia lymphoid (RHL) patients. Patients who met the following criteria were included: pathologically confirmed diagnosis of DLBCL; with complete clinical data and follow-up information. Exclusion criteria contained: with other malignancies; die from other causes. All patients received standard treatment according to NCCN Clinical Practice Guidelines [19]. Histological diagnoses were established according to the 2022 WHO classification [20]. Clinical data of newly diagnosed DLBCL patients were collected simultaneously.

### Cell culture and transfection

Human DLBCL cell lines LY1, LY3, U2932 and LY8 were cultured in Iscove-modified Dulbecco medium (IMDM, Gibco, Life Technologies, Carlsbad, CA, USA) with 10% fetal bovine serum (HyClone, Logan, UT, USA). PBMCs were obtained from two healthy donors, and isolated by the Ficoll-Hypaque density gradient centrifugation method. All cell lines were incubated in an atmosphere containing 5% CO_2_ at 37℃. All cells were examined for short tandem repeat (STR) and mycoplasma infection periodically. Green fluorescent protein (GFP) labeled lentiviral vector either eIF3a-konckdown or empty was purchased from Genechem (Shanghai, China). LY1 and LY8 cell lines were transfected with lentivirus according to the manufacturer’s instructions and screened with 5 µg/mL puromycin (Amresco, USA). The infection efficiency was assessed by qRT-PCR and western blot.

### Quantitative real-time PCR

Total RNA was extracted by RNAiso Plus (TaKaRa, Dalian, China), according to the manufacturer’s instruction, and cDNA was synthesized by reverse transcription reagent (TaKaRa). We used SYBR Green Master Mix (TaKaRa) in LightCycler 480II (Roche, Basel, Switzerland) to achieve amplification reaction. The eIF3a specific primers (Biosune, Shanghai, China) were as follows: forward, 5’-AAAACAACACCATCCTCCGC-3’ and reverse 5’-AACTCAGGGTCCGAGAAGTG-3’. The reference gene is GAPDH (TaKaRa), for-ward, 5’-GCACCGTCAAGGCTGAGAAC-3’ and reverse 5’-TGGTGAAGAC-GCCAGTGGA-3’. The quantification of mRNA was calculated using the 2-ΔΔCt method [21]. Real-time PCR for each gene of each cDNA sample was assessed in triplicate.

### Western blot

Proteins were extracted using radio-immunoprecipitation assay buffer (RIPA, Shenergy Biocolor, Shanghai, China) and 1% phenyl methyl sulfonyl fluoride (PMSF, Shenergy Biocolor), and protein concentration was detected using the BCA assay (Shenergy Biocolor). A total of 30 µg protein extract from every sample was then electrophoretically separated on a 7.5% polyacrylamide gel (Bio-Rad, USA) and transferred to PVDF membranes (Millipore, Billerica, MA, USA), which were blocked in 5% skimmed milk for 1 h. The blots were cut before hybridisation with antibodies, then incubated in primary antibody overnight at 4℃. The PVDF membranes were incubated in an HRP-conjugated secondary antibody (Zhongshan Goldenbridge, Beijing, China, 1:5000 dilution) for 1 h. Ultimately, the blots were detected using the electro-chemiluminescence kit (Millipore) by Amersham Imager 680 imaging system (General Electric, USA). The primary antibodies included eIF3a (ab128996, Abcam, Cambridge, UK, 1:2000 dilution), Cyclin D1 (26939-1-AP, Proteintech, USA, 1:5000 dilution), CDK4 (11026-1-AP, Proteintech, 1:1000 dilution), CDK6 (14052-1-AP, Proteintech, 1:1000 dilution) and GAPDH (TA309157, Zhongshan Goldenbridge, 1:1000 dilution). Image J software 1.44 was used to quantify protein band signals.

### Immunohistochemistry (IHC)

The paraffin-embedded tissues were sliced into 4 μm thick and then deparaffinized and hydrated. Antigen retrieval was performed using a 1 × EDTA antigen retrieval solution (Solarbio, Beijing, China). Endogenous peroxidase was blocked by 3% hydrogen peroxide and slides were incubated in goat serum (Solarbio) to block non-specific binding. The specimens were then incubated with anti-eIF3a antibody (ab128996, Abcam, 1:100 dilution) overnight at 4℃. Slides were incubated at 37℃ for 30 min, then handled with goat anti-rabbit biotinylated antibody and incubated with strept avidin-horseradish peroxidase complex (SABC). Finally, a diaminobenzidine (DAB) kit was used to stain the specimens, and hematoxylin was used to counterstain. Two independent observers who were blinded to patient’s clinical data scored the staining results. Five microscopic fields were observed at × 400 magnification. We calculated the IHC score by multiplying the expression level (0, 0–19%; 1, 20–49%; 2, 50–69%; 3, 70–100%) and staining intensity (0, negative; 1, weak; 2, moderate; 3, strong) of tumor cells. Scores of 0–3 were considered as negative expression, and 4–9 as positive expression [22].

### Cell proliferation assay

Cell proliferation was assessed using the cell counting kit-8 assay (CCK-8, Dojindo, Japan). LY1 and LY8 cells were seeded in 96 well plates at a 10,000 cells/well density for 24–96 h. After that, the cells were incubated with CCK-8 at 10 µL/well for 2 h at 37℃. Then, the cell proliferation was calculated by an absorbance value at 450 nm using Multiskan GO Microplate Reader (Thermo Scientific, Rockford, IL, USA).

### Apoptosis analysis

Annexin V-PE/7AAD apoptosis detection kit (BD Biosciences, Bedford, MA, USA) was employed to detect cell apoptosis. DLBCL cells with indicated treatments were harvested and resuspended in 1× binding buffer, followed by the addition of 5 µL of Annexin V-PE and 5 µL of 7-AAD. After gentle vibration and incubation for 15 min at room temperature in the dark, cells were subjected to the flow cytometry (FACS navios flow cytometer, Beckman Coulter, CA, USA) according to the manufacturer’s instructions.

### Identification and analysis of DEGs between two clusters based on eIF3a expression

The mRNA sequencing data of DLBCL patients in GSE23501 were employed to identify DEGs between two clusters based on eIF3a expression, with absolute log_2_ fold change (|log_2_FC|) > 1 and Wilcoxon test P value < 0.05. GO analysis of DEGs was performed via “clusterProfiler” and shown through “ggplot2” R package.

### Immune profile evaluation between two clusters based on eIF3a expression

22 immune cells of DLBCL samples in GSE23501 was estimated with “CIBERSORT” package in R. The discrepancy of several vital immune checkpoints between the two groups was calculated through “vioplot” and “ggplot2” packages in R to compare the immunity in tumor environment.

### Exploration of eIF3a-related mutation profile and potential drugs in DLBCL

Somatic mutation data such as variant classification, variant type of TCGA-DLBCL (*n* = 47) was employed for mutation analysis through “maftools” R package. All these samples were histologically diagnosed as DLBCL. Oncoplot was used to show the mutation profile of DEGs. Fisher’s exact test was calculated and displayed by correlation heatmap, and *P* < 0.05 was defined to have significance. Subsequently, all 47 samples were divided into two groups based on DEGs mutation. Mutational alteration of several vital pathways and potential druggable categories between DEGs mutation group and non-mutation group were also explored through “drugInteractions” algorithm.

### Pan-cancer analysis of eIF3a

Sequencing dataset from UCSC were employed to perform pan-cancer analysis of eIF3a. Differential expression of eIF3a between normal and tumor samples in other 34 types of tumors were calculated by Wilcoxon test and *P* < 0.05 was considered to be significant. The prognostic value of eIF3a was then calculated in 44 tumors from UCSC and previous study [23].

### Chemosensitivity analysis

Samples in GSE23501 were divided into two groups based on eIF3a expression, the “oncoPredict” R package was utilized to assess half of the maximum inhibitory concentration (IC50) of 198 kinds of drugs from Genomics of Drug Sensitivity in Cancer (http://www.cancerrxgene.org/). “ggpurb” R package was used to show the difference of IC50 in the two groups.

### Statistical analysis

The data of cell experiments were expressed as mean ± standard deviation, and SPSS 20.0 (SPSS Inc., Chicago, IL, USA) and GraphPad Prism 7.0 (San Diego, CA, USA) were used for statistical analysis. The t-test or one-way analysis of variance (ANOVA) was used to assess the differences between quantitative variables. The chi-square test was used to analyze the association between clinical data and eIF3a expression in DLBCL patients. Kaplan-Meier analysis was performed to assess the prognostic role of eIF3a expression in DLBCL patients. All experiments were conducted at least 3 times. Statistical significance was considered when *P* < 0.05 (* *P* < 0.05, ** *P* < 0.01, *** *P* < 0.001, **** *P* < 0.0001).

## Results

### eIF3a expression was upregulated in DLBCL and correlated with worse prognosis

**Fig. 1:**
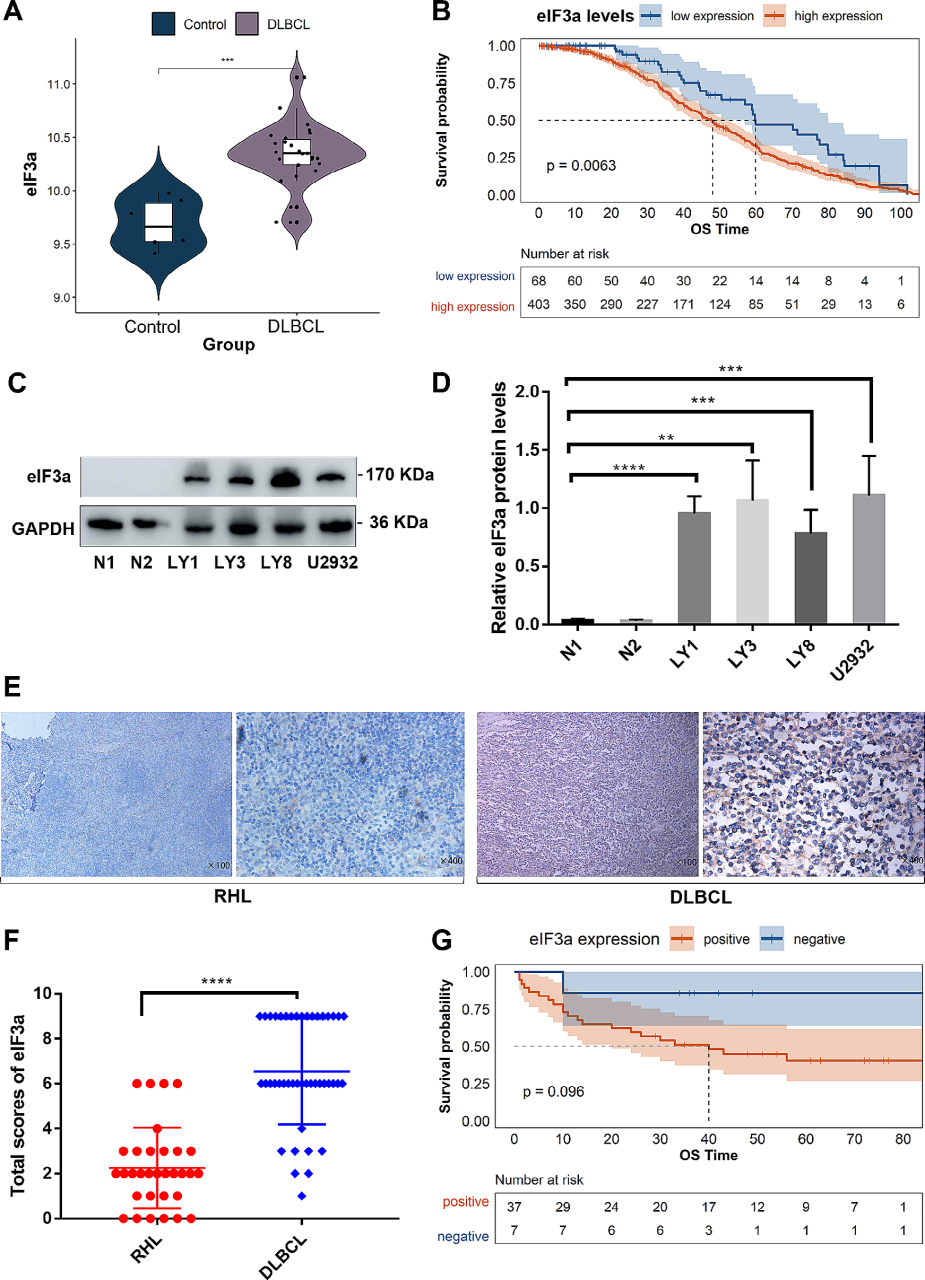
The expression of eIF3a was upregulated in DLBCL and correlated with worse prognosis. (**A**) The expression levels of eIF3a in DLBCL and healthy control groups from GSE25638. (**B**) Kaplan-Meier curves for OS of DLBCL patients with high- or low- expressed eIF3a levels in GSE31312 (*P* = 0.0063). (**C**, **D**) The expression levels of eIF3a protein in DLBCL cell lines (LY1, LY3, LY8 and U2932) and PBMCs from healthy donors. The results of triplicate experiments were shown as the mean ± SD. (**E**, **F**) Compared with RHL, the expression level of eIF3a was significantly increased in DLBCL tissues. Original magnification, ×100 and ×400. (**G**) Kaplan-Meier survival analysis showed shorter OS in patients with positive expression of eIF3a. **P < 0.01, *** *P* < 0.001, **** *P* < 0.0001

To illuminate the expression level of eIF3a in DLBCL, we firstly compared eIF3a expression between DLBCL and healthy controls in GSE25638, and found obviously high expression of eIF3a mRNA in tumor tissue (Fig. 1A). DLBCL patients in GSE31312 dataset were divided into two groups based on eIF3a expression (Figure S2A), patients with high eIF3a expression had worse prognosis compared to these with low level (Fig. 1B, *P* = 0.0063). We also used another large dataset GSE181063 (*n* = 1037) to verify the prognostic value of eIF3a, demonstrating high eIF3a expression was significantly associated to inferior prognosis (Figures S2C, D, *P* = 0.0018). The expression of eIF3a protein was significantly increased in DLBCL cells compared with PBMCs from healthy donors (Fig. 1C–D), but there were no obvious differences between different cell lines of ABC subtypes (LY3 and U2393) and GCB subtypes (LY1 and LY8). We also found that eIF3a mRNA levels had no significant differences among ABC, GCB and unclassified subtypes (Figure S2B). IHC results demonstrated that the expression of eIF3a protein was significantly higher in DLBCL tissues than in RHL (Fig. 1E, F). The positive rate of eIF3a was 84.09% (37 of 44) in DLBCL tissues whereas 15.63% (5 of 32) in RHL.

Next, we analyzed the relationship between eIF3a expression and clinical characteristics in DLBCL patients (Table 1). Positive expression of eIF3a was closely associated with advanced Ann Arbor stage (*P* = 0.044) and high international prognostic index (IPI) score (*P* = 0.011). Kaplan-Meier survival curve revealed that DLBCL patients with positive eIF3a expression displayed shorter OS than those negative, though there was no statistical significance (Fig. 1G, *P* = 0.096). This could be mainly attributed to the small-scale of cohort. These results found that eIF3a expression was upregulated in DLBCL and correlated with worse prognosis, indicating the potential of eIF3a as prognostic biomarker in DLBCL patients.

**Table 1:** The correlation between eIF3a expression and clinical characteristics of DLBCL patients

Characteristics	No. of patients	eIF3a expression		*P* value
		Positive	Negative	
**Age(years)**				
≤ 60 > 60	17 27	13 24	4 3	0.501
**Gender**				
Male Female	26 18	22 15	4 3	1.000
**Ann Arbor stage**				
I or II III or IV	14 30	9 28	5 2	**0.044**
**Albumin**				
< 40 g/L ≥ 40 g/L	26 18	24 13	2 5	0.170
**Serum LDH**				
Normal Elevated	20 24	15 22	5 2	0.275
**IPI score**				
0–2 3–5	16 28	10 27	6 1	**0.011**
**Anemia**				
YES NO	14 30	12 25	2 5	1.000
**Fibrinogen**				
≤ 4 g/L > 4 g/L	24 20	19 18	5 2	0.572
**LMR**				
≤ 2.25 > 2.25	16 28	12 25	4 3	0.413

LDH: lactate dehydrogenase, IPI: international prognostic index, LMR: Ratio of lymphocytes to monocytes

### eIF3a promoted the growth of DLBCL cells

**Fig. 2:**
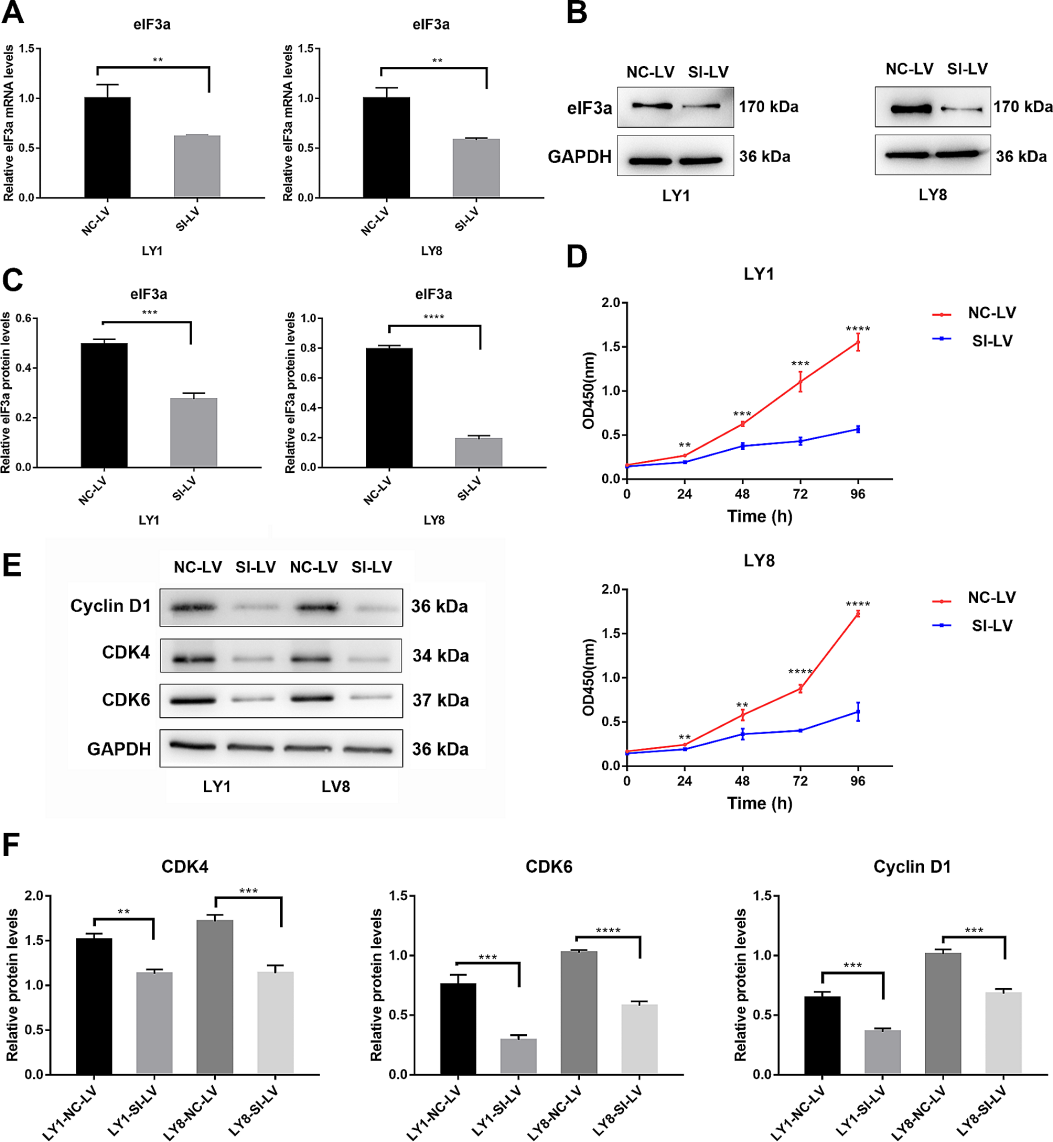
eIF3a promoted the growth of DLBCL cells. (**A**) Relative expression of eIF3a mRNA was confirmed by qRT-PCR. (**B**, **C**) DLBCL cells transfected with SI-LV displayed significantly decreased expression of eIF3a protein than those transfected with NC-LV. (**D**) Cell proliferation was evaluated by CCK8 assays. The proliferation efficiency of DLBCL cells was markedly decreased in DLBCL cells with eIF3a knock down. (**E**, **F**) Knockdown of eIF3a reduced the expression of proliferation-related proteins. The results of triplicate experiments were shown as the mean ± SD. **P < 0.01, *** *P* < 0.001, **** *P* < 0.0001

To further explore the biological function of eIF3a, human DLBCL cells LY1 and LY8 were stably transfected with either negative control lentiviral vector (NC-LV) or eIF3a stable interference lentiviral vector (SI-LV). Two types of SI-LV were used, and one with superior knockdown efficacy was used for further experiments. Effective silencing of eIF3a in DLBCL cells was confirmed by qRT-PCR and western blot (Figs. 2A-C, S1A, B). Cell proliferation rates of LY1 and LY8 were evaluated by CCK8 assay, and the results showed that the proliferation rates of DLBCL cells transfected with SI-LV were significantly reduced than those transfected with NC-LV (Fig. 2D). Furthermore, we detected the expression level of cyclin D1 (CCND1), cyclin-dependent kinase 4 (CDK4), and cyclin-dependent kinase 6 (CDK6) by western blot to deeper evaluate the regulation roles of eIF3a on proliferation-related proteins in DLBCL cells. As expected, after reducing eIF3a expression, these three proteins levels were significantly decreased (Fig. 2E, F), suggesting eIF3a might promote the growth of DLBCL cells via regulating cell proliferation and cell cycle.

### eIF3a knockdown induced apoptosis of DLBCL cells

**Fig. 3:**
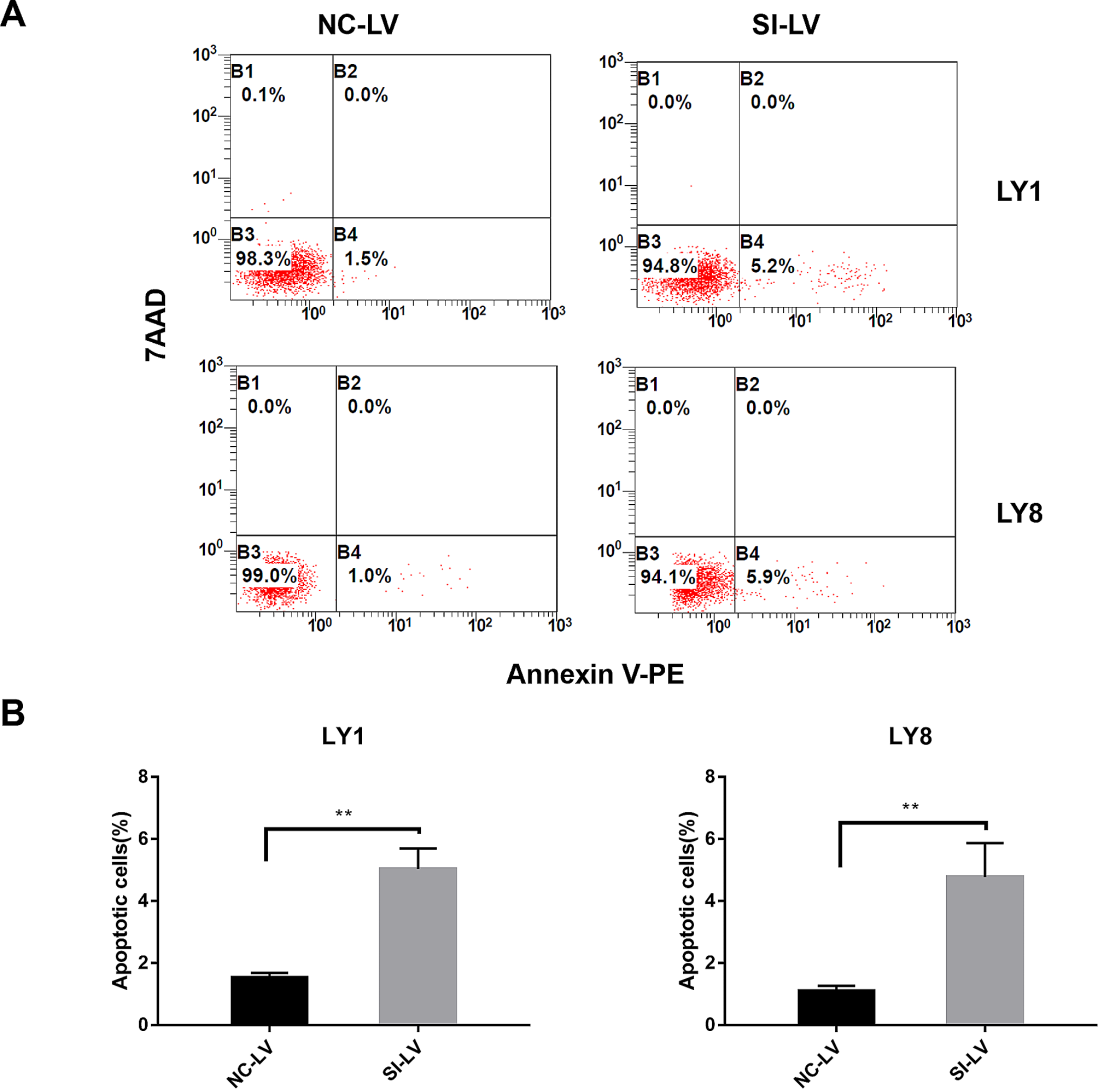
The knockdown of eIF3a induced the apoptosis of DLBCL cells. (**A**, **B**) The knockdown of eIF3a resulted in increased apoptosis rates in DLBCL cells (LY1 and LY8 cells) based on flow cytometric analysis with Annexin V-PE/7AAD staining. The results of triplicate experiments were shown as the mean ± SD. **P < 0.01

The Annexin V-PE/7AAD apoptosis assay was performed to investigate the regulatory role of eIF3a on cell apoptosis in DLBCL. The flow cytometry analysis showed that the apoptosis rates in both LY1 cells (5.03 ± 0.67% in SI-LV group vs. 1.53 ± 0.15% in NC-LV group, *P* = 0.0075) and LY8 cells (4.77 ± 1.10% in SI-LV group vs. 1.10 ± 0.17% in NC-LV group, *P* = 0.0047) were increased after eIF3a knockdown (Fig. 3). These results indicated that eIF3a might serve an important role in the apoptosis of DLBCL cells.

### Function analysis of DEGs between low- and high-expressed eIF3a clusters

**Fig. 4:**
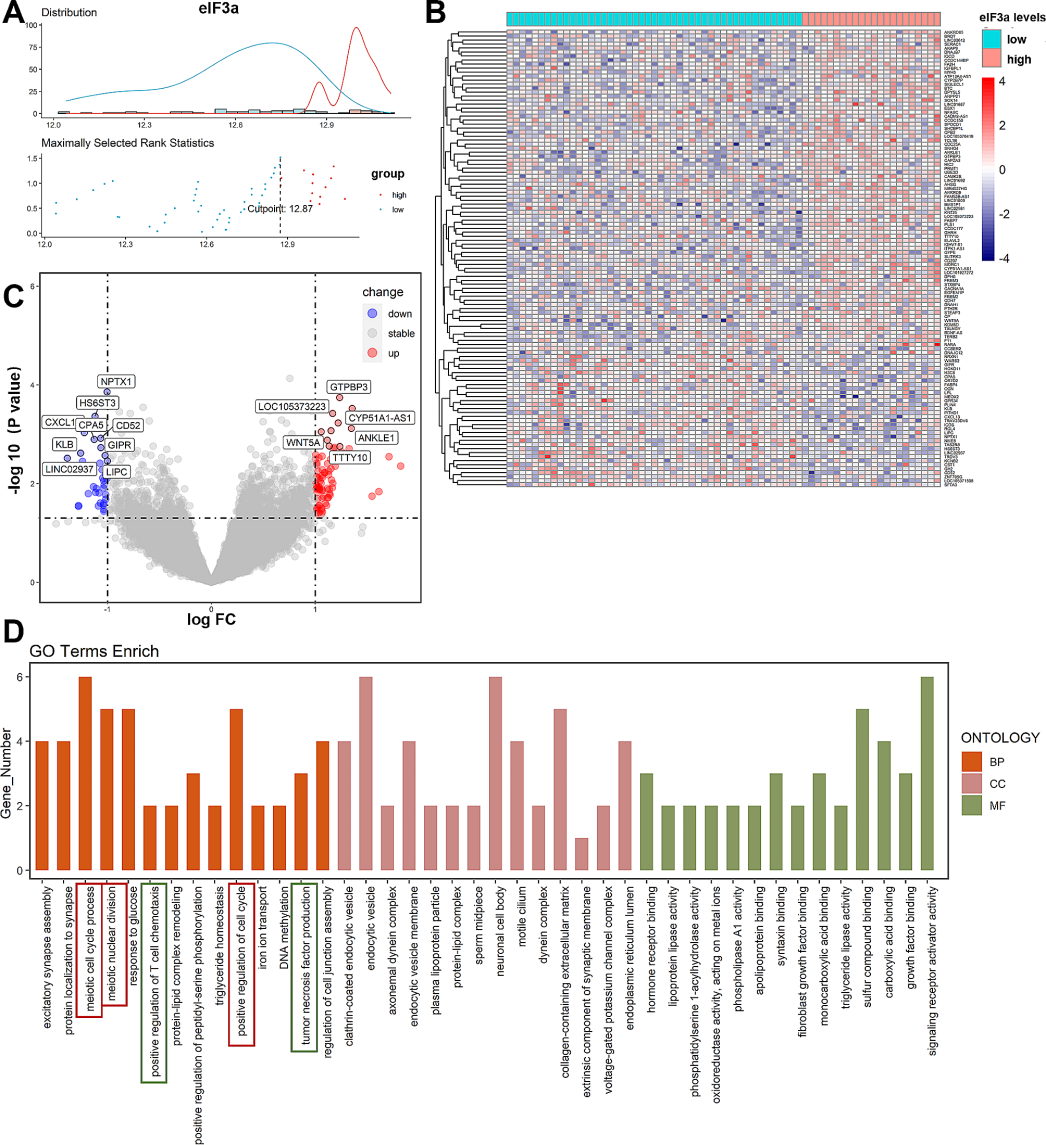
Function analysis of differentially expressed genes between low- and high-expressed eIF3a clusters. (**A**) The optimum cut value of eIF3a expression in GSE23501. The green line represented “distribution” and red line represented “density”. (**B**, **C**) Differentially expressed genes between low- and high-expressed eIF3a clusters which were identified based on|log_2_FC| > 1 and *P* < 0.05. (**D**) The most significant GO enrichment pathways focused on differentially expressed genes

To further explore the biological function of eIF3a in DLBCL development, we analyzed mRNA sequencing data of GSE23501 to identify DEGs between two clusters based on eIF3a expression. As (Fig. 4A) showed, eIF3a ≥ 12.87 were defined as high-expressed while < 12.87 were low-expressed based on the maximum rank statistic through “surv_cutpoint” algorithm in R. 114 differentially expressed mRNAs between the two groups were identified (Fig. 4B, C). Functional enrichment analysis showed that these DEGs had a close linkage to cell cycle process such as meiotic cell cycle process and meiotic nuclear division (Fig. 4D). Besides, immune related pathways were also enriched, indicating that eIF3a possessed a potential relationship to tumor immunity in DLBCL.

### Immune profiling of DLBCL patients between two clusters based on eIF3a expression

**Fig. 5:**
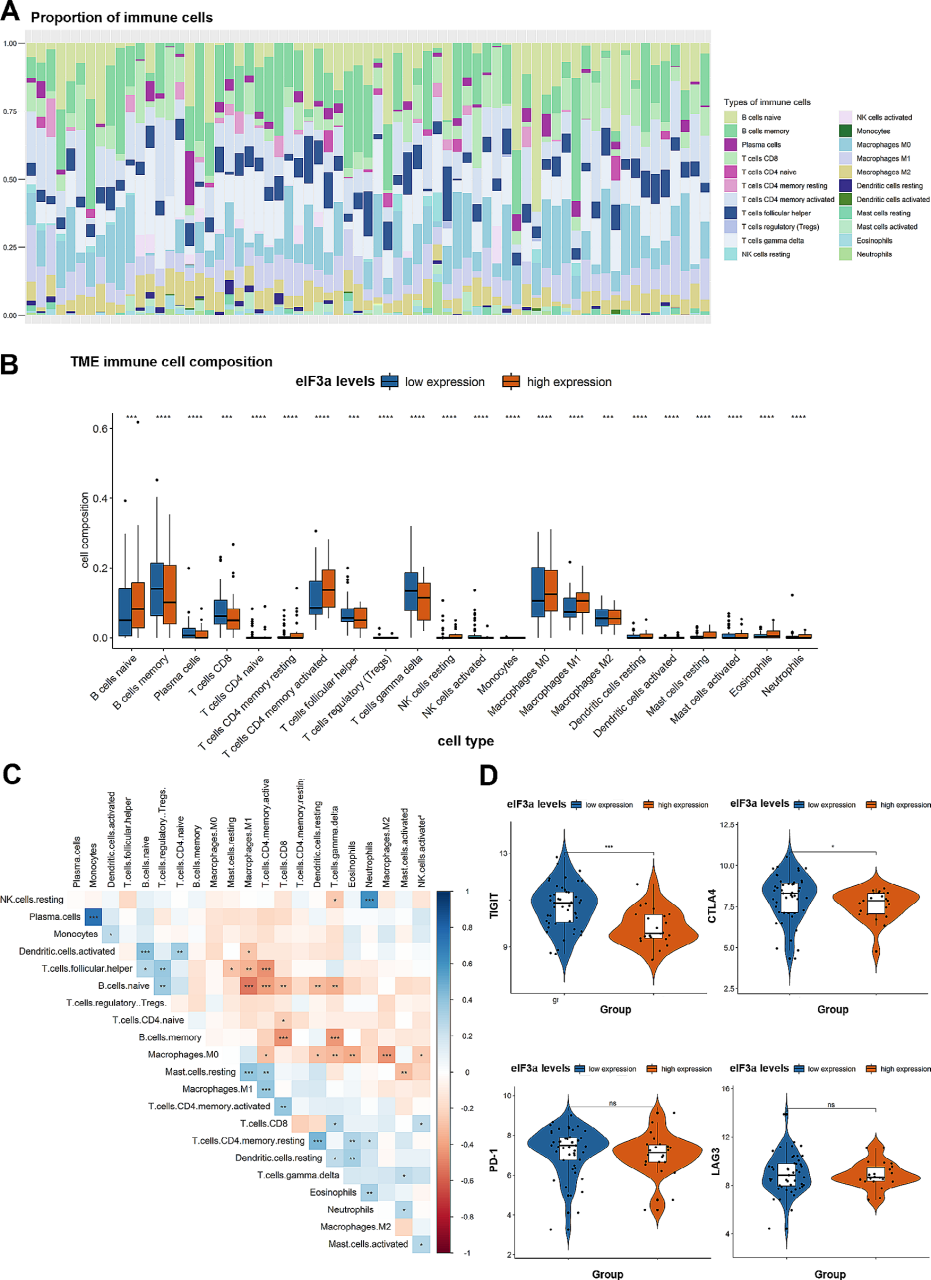
Immune profiling of DLBCL patients between the two groups based on eIF3a levels. (**A**) The abundance of 22 different immunocytes in DLBCL samples from GSE23501. Each column represented a sample; and the different colors represented different immune cells. (**B**) The differences of 22 types of immune cells between low- and high-expressed eIF3a groups. (**C**) The correlations and interactions between 22 immune cells. Positive correlations are marked with blue and negative correlations with red. (**D**) The expression level of TIGIT, CTLA4, PD-1, LAG3 in the two groups. *P < 0.05, **P < 0.01, *** *P* < 0.001, **** *P* < 0.0001

According to the findings above, we speculated that eIF3a might played critical roles in immune response and regulation of tumor environment, and further validated it. GSE23501 were subsequently utilized to explore immune profiling of DLBCL patients. 22 types of immunocytes were calculated through “CIBERSORT” package in R to identify immune components (Fig. 5A, B). Notably, several cytotoxic immunocytes such as CD8 positive T cells and γ/δ T cells were verified to be significantly accumulated in low-expressed eIF3a cluster (Fig. 5B, *P* < 0.05). Besides, landscape of immunocytes interactions in DLBCL patients was exhibited by the correlation coefficient heatmap, both naïve and memory B cells had significant responses to other immunocytes, especially T cells (Fig. 5C). Then we compared the expression levels of immune checkpoints containing TIGIT, CTLA4, PD-1 and LAG3 between two subgroups. As Fig. 5D indicated, high expression of TIGIT and CTLA4 were observed in the low-expressed eIF3a cluster (*P* < 0.05), indicating these patients may tend to benefit from immunotherapy. Overall, our results demonstrated that low-expressed eIF3a was related to more activated immune microenvironment such as immune response and inflammatory cells activation, which might partially explain the mechanism underlying the prognostic role of eIF3a in DLBCL patients.

### Mutation characteristics of eIF3a and DEGs in DLBCL

**Fig. 6:**
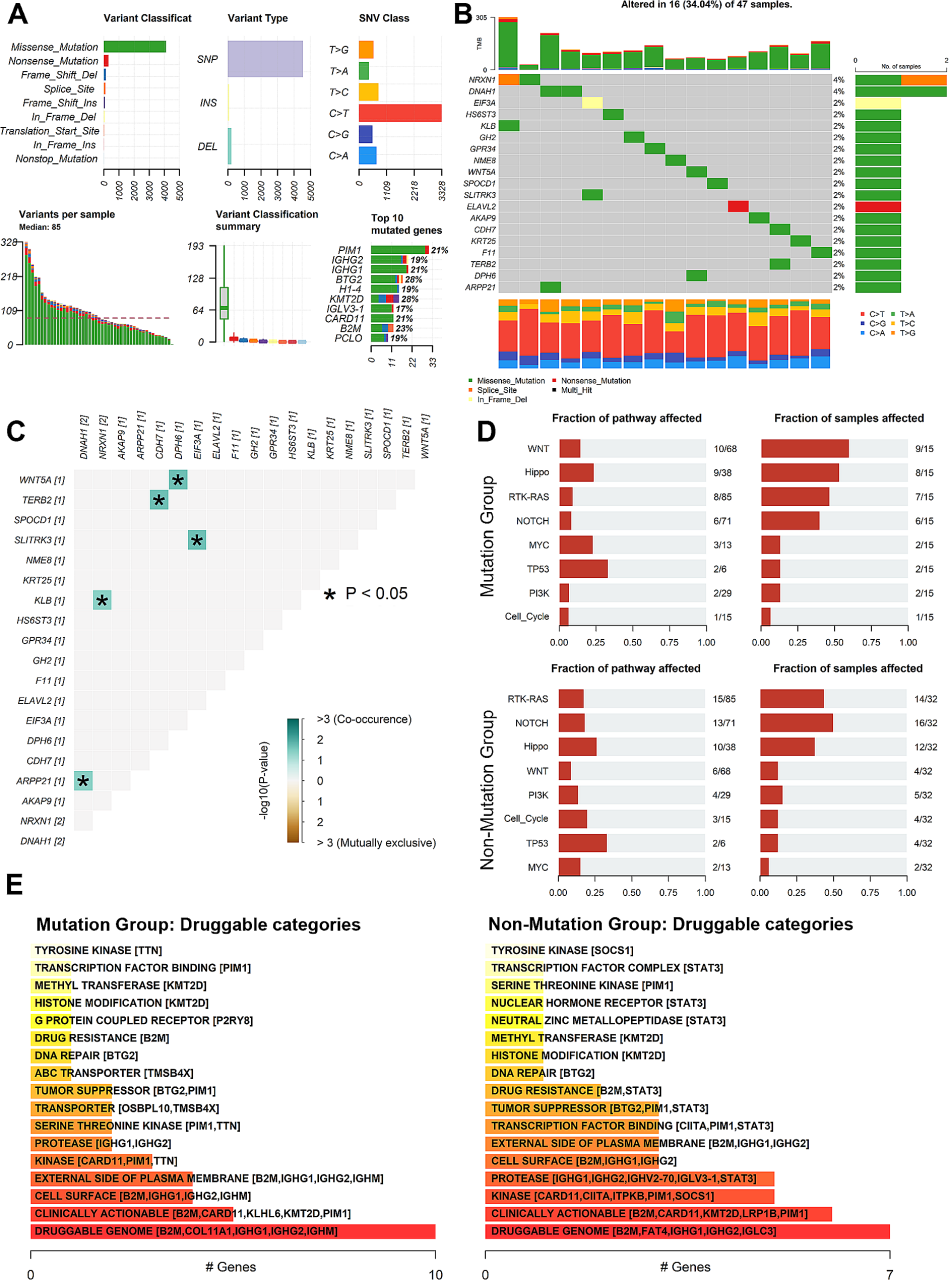
Mutation characteristics of eIF3a and DEGs in DLBCL. (**A**) Mutation generalization of TCGA-DLBCL datasets (*n* = 47). (**B**) A mutation oncoplot of eIF3a and DEGs in DLBCL. (**C**) The correlation heatmap of co-occurrence and mutually exclusive mutations of eIF3a and DEGs based on Fisher’s exact test. (**D**) Mutation rate and samples in some vital signal pathways between mutation group and non-mutation group. (**E**) Predicted druggable categories between mutation group and non-mutation group. When enriched genes were more than 5, only the first 5 were displayed

Somatic mutation profile data (*n* = 47) was applied to further investigate the genetic and biological role of eIF3a in DLBCL,   Figure 6A generalized the mutation pattern of the dataset. eIF3a and DEGs mutation were observed in 34.04% of all 47 samples, and eIF3a mutation rate were 2% (Fig. 6B). Among these DEGs, most mutations were found to be co-occurrence mutations, such as WNT5A and DPH6 (Fig. 6C). Subsequently, all 47 samples were divided into mutation group (*n* = 15) and non-mutation group (*n* = 32, included nonsense-mutation). Mutations in several signal pathway showed significant difference. The Hippo and Wnt pathway mutation rate were high in mutation group, while cell cycle pathway mutation rate were high in non-mutation group (Fig. 6D). Moreover, in mutation group, some genes were enriched in known drug targets including PIM1 and KMT2D (Fig. 6E). These findings suggested that eIF3a-related mutations may exhibit a promising capacity to predict drug sensitivity in DLBCL. Deeper experiments and further large-cohort clinical trials are urgently needed to evaluate the efficacy of these drugs.

### Pan-cancer analysis and chemosensitivity comparison of eIF3a

**Fig. 7:**
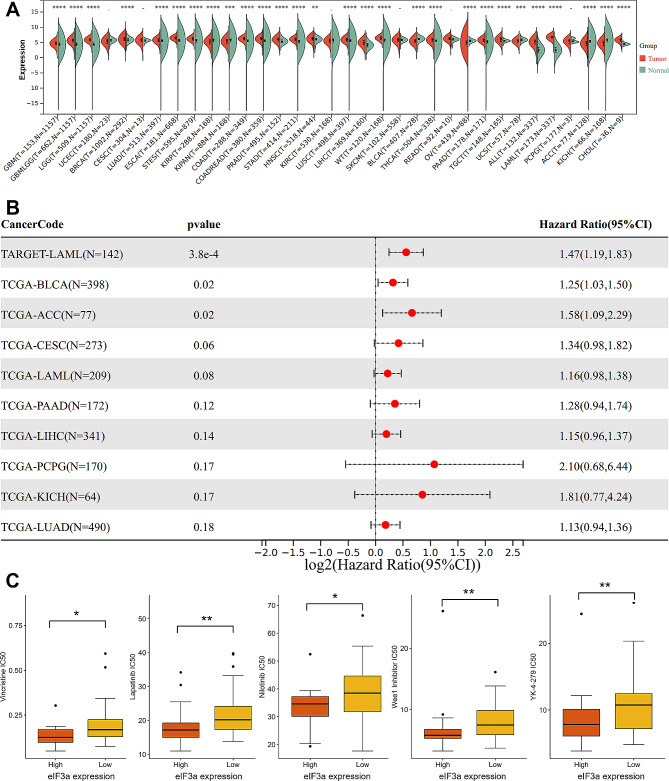
Pan-cancer analysis and chemosensitivity comparison of eIF3a. (**A**) The expression of eIF3a in 34 types of cancers based on standardized pan-cancer dataset from UCSC. (**B**) The top 10 most significant relationship between eIF3a expression and prognosis of tumor patients. (**C**) The differences of IC50 between different groups based on eIF3a expression. **P < 0.01, *** *P* < 0.001, **** *P* < 0.0001

To verify whether eIF3a was upregulated and associated with prognosis in other types of tumors, we further explored eIF3a expression in other 34 kinds of tumors. Among them, increased eIF3a expression were observed in 21 types of tumors, which included acute lymphoblastic leukemia (ALL) and acute myeloid leukemia (AML) (Fig. 7A, *P* < 0.05). Besides, (Fig. 7B) showed the top 10 most significant relationship between eIF3a expression and prognosis in tumor patients. High eIF3a expression were demonstrated to be correlated with worse outcome in 3 tumors including AML, bladder urothelial carcinoma (BLCA) and adrenocortical carcinoma (ACC). However, eIF3 expression in different tumors was not consistent, and there were 7 types of tumors with decreased expression of eIF3a (Fig. 7A). Further studies are still required to deeply clarify the biological role and molecular mechanism of eIF3a in the development and progression of tumors.

Chemosensitivity analysis showed that the differences of IC50 were significant in 24 among 198 drugs (Table S1, *P* < 0.05). Especially, 23 kinds of drugs were more sensitive to patients with high eIF3a expression, and only IAP-5620 was more sensitive to patients in the low-expression group. For example, IC50 of Vincristine, a common chemotherapeutic agent of many combination regimens for lymphoid malignancies, displayed an obvious difference in the two groups, indicating that high expression of eIF3a may be correlated with higher sensitivity to Vincristine in DLBCL (Fig. 7C).

## Discussion

In this study, we for the first time elucidated that eIF3a was upregulated in DLBCL tissues and cell lines, which was positively correlated with clinical features of DLBCL patients. DLBCL patients with positive eIF3a expression had shorter OS. eIF3a knockdown significantly inhibited cell proliferation and increased apoptosis rate in DLBCL cells. Moreover, 114 DEGs were identified in the two clusters based on eIF3a expression, which had a close linkage to cell cycle and tumor immune. Cytotoxic immunocytes were verified to be significantly decreased in high-expressed eIF3a clusters, consistently to poorer prognosis in DLBCL patients. The mutation characteristics of eIF3a and DEGs had the potential to predict drug sensitivity in DLBCL. eIF3a expression was also clarified to be associated with chemosensitivity to several anti-tumor drugs. These results indicated that eIF3a might become a promising prognostic biomarker and therapeutic target in DLBCL treatment.

Till now, the correlation between eIF3a expression and prognosis in tumors is controversial. Recent studies have shown that eIF3a was highly expressed in esophageal squamous cell carcinoma (ESCC), colorectal cancer and thyroid cancer (TC) [16–18], but lower in clear cell renal cell carcinoma (ccRCC) [24]. Moreover, eIF3a expression was also related to prognosis of tumor patients. In patients with ESCC, ccRCC, low-grade UBC and oral squamous cell carcinoma (OSCC), high levels of eIF3a were associated with favorable prognosis [11, 18, 24, 25]. Similarly, breast cancer patients with high eIF3a expression were more sensitive to anthracycline drugs and displayed better prognosis [26]. However, Haybaeck J and Mei C et al. found that higher expression of eIF3a was associated with high tumor proliferation rate, distant metastasis rate and worse prognosis of colorectal carcinoma [17, 27]. Correspondingly, our findings revealed that eIF3a expression was obviously increased in DLBCL, and has a close linkage with IPI score and Ann-Arbor stage, suggesting that eIF3a may be related to the progression of DLBCL. Kaplan-Meier analysis further proved the association between high expression of eIF3a and shorter OS in DLBCL patients. Overall, these findings suggested that eIF3a might serve as a promising biomarker for prognostic evaluation in DLBCL patients, which needs to be validated in larger cohort of tumor patients.

Published studies have demonstrated that eIF3a impacted tumor development through regulating several biological processes, including cell proliferation, apoptosis, colony formation and migration [11, 28]. One study reported that eIF3a overexpression resulted in a higher proliferation rate, enhanced clonogenicity, resistance to apoptosis, and malignant transformation of immortal fibroblasts [29]. Besides, in TC and HCC tissues, high level of eIF3a had correlation with acceleration of cell proliferation and apoptotic decrease [12, 16]. eIF3a knockdown was found to reverse the malignant phenotype of lung and breast cancer cells [30]. Downregulation of eIF3a also impaired the proliferation, colony formation, and migration of UBC cells [11]. Previous studies found that eIF3a participated in many tumor development processes, such as maintaining the stem cell-like characteristics, mitochondrial dysfunction and glycolysis activation in tumor cells, which further contributed to cell cycle and proliferation [31, 32]. Consistently, our results showed that eIF3a knockdown could cause the inhibited cell proliferation and enhanced cell apoptosis in DLBCL cell, indicating the key role of eIF3a in the development of DLBCL. The regulatory roles and molecular mechanisms of eIF3a on biological processes in tumor development need to be further clarified.

CCND1 has been identified as a G1 to S cell cycle regulator and about 2.1% of DLBCL patients with positive CCND1 expression, especially in younger male patients [33]. The association between CCND1 expression and prognosis remained controversial in DLBCL patients [33, 34]. Our study found that the CCND1 protein expression in DLBCL cell lines with eIF3a knockdown was significantly reduced, which suggested that reducing the expression of eIF3a would inhibit the proliferation of DLBCL cells via inducing cell cycle arrest. Interestingly, there was no statistical significance in CCND1 RNA level in our study. The possibilities for this difference in protein and RNA levels may be concluded that eIF3a impacted the translation process, which has been reported in other tumors [17].

Furthermore, CDKs are a group of protein kinases that play essential role in controlling cell division and cell cycle [35]. Among them, CDK4 and CDK6 have similar biochemical and biological characteristics, and both of them could promote the synthesis of D-type cyclins, thereby promoting the process of cell cycle. It has been confirmed that CDK4 and CDK6 are closely related to tumorigenesis [36, 37], and inhibition of CDK4/6 could bring an anti-tumor effect in several tumors [38, 39], including lymphoma [40, 41]. To date, the U.S. Food and Drug Administration (FDA) has approved three selective CDK inhibitors (palbociclib, ribociclib, and abemaciclib) for breast cancer, and 15 CDK4/6 inhibitors are in clinical trials [42]. Our study showed that knockdown of eIF3a reduced the CDK4 and CDK6 expression in DLBCL cells, suggesting that targeting eIF3a has a potential anti-tumor effect in DLBCL.

Apoptosis, a meaningful way to remove abnormal cells, is necessary for the body metabolism and homeostasis. Currently, many new drugs play a therapeutic role in lymphoma by promoting the apoptosis of tumor cells [43–45], such as LW-213, BM-1197, 1, 2-diazole. Previous studies have found that there was no correlation between eIF3a expression and p53, who participates in the intrinsic and extrinsic cell death pathways, indicating the eIF3a-related apoptosis may through p53-independent pathway [14]. Our results found that eIF3a knockdown could increase the apoptosis rate of DLBCL cells, indicating the regulatory role of eIF3a in cell apoptosis, and further studies are needed to explore underlying mechanism.

Point mutations of certain genes have been demonstrated to affect cell behavior and tumor progression [46, 47]. For example, eIF3a mutation was found to be related to acquired chemotherapy resistance in lung cancer [48], and a low-frequency missense variant in SPOCD1 was associated with reduced risk of gastric cancer [49]. In our study, eIF3a and DEGs mutation were widely observed in DLBCL samples, and predicted drug sensitivity, suggesting that deep exploration of these mutations could further reveal the heterogeneity, and provide novel targets for precision treatment in DLBCL.

Previous studies have widely demonstrated that immune function was restricted in malignancies, such as inferior immune status and aberrant immune checkpoints, which was closely related to prognosis [50, 51]. Immune profiling analysis found that several cytotoxic immunocytes such as CD8^+^ T cells and γ/δ T cells were verified to be significantly accumulated in low-expressed eIF3a clusters, which were associated with improved survival outcome [52, 53]. Moreover, we found less enriched immunocytes and more inactive components in high eIF3a subgroup, indicating that these patients may tend to be resistant to immunotherapeutic interventions. Immune checkpoints such as TIGIT and CTLA4 were denser in low eIF3a group, indicating that eIF3a may not only provide therapeutic target for DLBCL patients but also act as potential predictors of immunotherapy responses.

High heterogeneity of DLBCL forced deeper understandings of individualized treatment regime. We performed chemosensitivity analysis in different groups based on eIF3a expression, finding that eIF3a expression had a close linkage to several drugs including Vincristine and novel inhibitors (Wee1 inhibitor, YK-4-279, MK-1775 et al.). Previous studies have revealed that Wee1 inhibitor had great anti-tumor potential together with other G2/M arresting or DNA damaging therapeutic compounds in DLBCL [54]. YK-4-279 was also found to exert anti-lymphoma activity via blocking the protein-protein interaction with RNA helicases [55]. These results indicated that eIF3a has great potential to act as a biomarker for chemosensitivity in DLBCL.

In conclusion, our findings firstly identified that eIF3a was highly expressed in DLBCL, which was correlated with clinical characteristics and worse prognosis. eIF3a knockdown could inhibit the growth of DLBCL cells through regulating cell proliferation and apoptosis. Further function and immune analysis revealed that high eIF3a expression tends to be associated with immunosuppressive status and poorer prognosis. eIF3a and DEGs mutations were found to be correlated to chemosensitivity of some anti-tumor drugs and vital signal pathways. eIF3a expression was also demonstrated to be associated with chemosensitivity to several anti-tumor drugs. These findings suggest that eIF3a might serve as an indicator of prognosis and drug sensitivity for DLBCL treatment. Nevertheless, further researches are needed to explore the molecular mechanism of eIF3a in DLBCL development, which might provide more novel ideas for DLBCL treatment.

### Electronic supplementary material

Below is the link to the electronic supplementary material.

Supplementary Material 1

Supplementary Material 2

## Data Availability

More requirements of data are available from the corresponding author on reasonable request.

## Abbreviations

ACC: adrenocortical carcinoma
ALL: acute lymphoblastic leukemia
AML: acute myeloid leukemia
BLCA: bladder urothelial carcinoma
CCND1: Cyclin D1
ccRCC: clear cell renal cell carcinoma
CDK4: cyclin-dependent kinase 4
CDK6: cyclin-dependent kinase 6
DAB: diaminobenzidine
DEGs: differentially expressed genes
DLBCL: diffuse large B-cell lymphoma
eIF3: eukaryotic initiation factor 3
eIF3a: eukaryotic initiation factor 3a
ESCC: esophageal squamous cell carcinoma
FDA: Food and Drug Administration
GEO: Gene Expression Omnibus
GFP: green fluorescent protein
GO: Gene Ontology
HCC: hepatocellular carcinoma
IC50: half of the maximum inhibitory concentration
IHC: immunohistochemistry
IPI: international prognostic index
LDH: serum lactate dehydrogenase
|log2FC|: absolute log2 fold change
NC-LV: negative control lentiviral vector
OS: overall survival
OSCC: oral squamous cell carcinoma
PBMCs: peripheral blood mononuclear cells
PBS: phosphate buffer solution
RHL: reactive hyperplasia lymphoid
SABC: strept avidin-horseradish peroxidase complex
SI-LV: stable interference lentiviral vector
STR: short tandem repeat
TC: thyroid cancer
TCGA: The Cancer Genome Atlas
TNF: tumor necrosis factor
UBC: urinary bladder cancer
