# Supplementary material for: Eukaryotic initiation factor 3a promotes the development of diffuse large B-cell lymphoma through regulating cell proliferation
Source: BMC Cancer. 2024 Apr 8;24:432. doi: 10.1186/s12885-024-12166-0 (PMC11003032; doi:10.1186/s12885-024-12166-0)
Supplement: Supplementary file 1 — Supplementary Material 1 [file 12885_2024_12166_MOESM1_ESM.docx]

**Supplementary Tables**

**Table S1. The differences of IC50 of 198 drugs in the two groups based on eIF3a expression**

| **Drugs** | **Difference of IC50 ^[1]^** | **P value** |
| --- | --- | --- |
| Camptothecin_1003 | -0.01337 | 0.230557221 |
| **Vinblastine_1004** | **-0.0017** | **0.030269977** |
| Cisplatin_1005 | -0.57082 | 0.677881569 |
| Cytarabine_1006 | -0.20699 | 0.454219006 |
| Docetaxel_1007 | -0.00173 | 0.103676641 |
| **Gefitinib_1010** | **-3.40721** | **0.006870485** |
| Navitoclax_1011 | -1.44051 | 0.320915725 |
| **Vorinostat_1012** | **-0.46042** | **0.034557237** |
| **Nilotinib_1013** | **-3.86417** | **0.03934337** |
| Olaparib_1017 | -2.13727 | 0.763971692 |
| Axitinib_1021 | -0.45237 | 0.595745553 |
| AZD7762_1022 | -0.09529 | 0.197241512 |
| SB216763_1025 | 8.55575 | 0.296441004 |
| KU-55933_1030 | -1.18675 | 0.622629968 |
| **Afatinib_1032** | **-1.02511** | **0.03934337** |
| Staurosporine_1034 | 0.003527 | 0.3736458 |
| PLX-4720_1036 | -13.1532 | 0.05542844 |
| NU7441_1038 | -0.00254 | 0.984712167 |
| Doramapimod_1042 | -0.20653 | 0.974523006 |
| **Wee1 Inhibitor_1046** | **-1.50145** | **0.008406023** |
| Nutlin-3a (-)_1047 | -12.9918 | 0.240759873 |
| Mirin_1048 | -7.26375 | 0.461992449 |
| PD173074_1049 | -6.92403 | 0.240759873 |
| ZM447439_1050 | -1.43374 | 0.098251687 |
| Alisertib_1051 | -0.99245 | 0.197241512 |
| RO-3306_1052 | -0.40946 | 0.211068157 |
| MK-2206_1053 | -3.51865 | 0.058865239 |
| Palbociclib_1054 | -4.30514 | 0.070231586 |
| Dactolisib_1057 | -0.01894 | 0.44651868 |
| Pictilisib_1058 | -0.6607 | 0.290519676 |
| AZD8055_1059 | 0.017339 | 0.595745553 |
| PD0325901_1060 | -0.01284 | 0.933823592 |
| Obatoclax Mesylate_1068 | 0.06859 | 0.526735766 |
| 5-Fluorouracil_1073 | 2.014359 | 0.725281014 |
| Dasatinib_1079 | 0.160513 | 0.501934878 |
| Paclitaxel_1080 | -0.00778 | 0.124523866 |
| Crizotinib_1083 | -1.15826 | 0.485745471 |
| Rapamycin_1084 | -0.00582 | 0.734893498 |
| Sorafenib_1085 | -1.29231 | 0.106475532 |
| BI-2536_1086 | -0.13587 | 0.423861861 |
| Irinotecan_1088 | -2.51099 | 0.112249754 |
| Oxaliplatin_1089 | -0.95758 | 0.44651868 |
| BMS-536924_1091 | -1.84166 | 0.064344881 |
| GSK1904529A_1093 | -3.83104 | 0.401881518 |
| Tozasertib_1096 | -0.99494 | 0.394707076 |
| PF-4708671_1129 | 0.238598 | 0.622629968 |
| PRIMA-1MET_1131 | -2.49839 | 0.803251416 |
| Erlotinib_1168 | -0.59827 | 0.380588899 |
| Niraparib_1177 | 0.827139 | 0.423861861 |
| **MK-1775_1179** | **-0.2829** | **0.026440981** |
| Dinaciclib_1180 | -0.00285 | 0.431339592 |
| Gemcitabine_1190 | 0.02775 | 0.964337936 |
| Bortezomib_1191 | -0.0002 | 0.526735766 |
| GSK269962A_1192 | -0.28449 | 0.416459313 |
| SB505124_1194 | 0.626974 | 0.066260897 |
| **Tamoxifen_1199** | **-4.80559** | **0.011492711** |
| Fulvestrant_1200 | -0.96095 | 0.081001961 |
| EPZ004777_1237 | -14.568 | 0.064344881 |
| **YK-4-279_1239** | **-2.00802** | **0.009465584** |
| Daporinad_1248 | -0.00065 | 0.327231779 |
| BMS-345541_1249 | -3.30553 | 0.127747651 |
| AZ960_1250 | -0.66871 | 0.240759873 |
| Talazoparib_1259 | -3.23472 | 0.314678599 |
| XAV939_1268 | -2.58584 | 0.290519676 |
| Trametinib_1372 | 0.211347 | 0.501934878 |
| **Dabrafenib_1373** | **-12.1161** | **0.029271313** |
| **Temozolomide_1375** | **-32.0185** | **0.024686142** |
| AZD5438_1401 | -0.69661 | 0.501934878 |
| **IAP_5620_1428** | **19.72786** | **0.049047677** |
| AZD2014_1441 | -0.02551 | 0.95415859 |
| AZD1208_1449 | -12.3995 | 0.13780252 |
| AZD1332_1463 | -2.15531 | 0.416459313 |
| Ruxolitinib_1507 | -3.96043 | 0.256643431 |
| **Linsitinib_1510** | **-7.85301** | **0.031297274** |
| Epirubicin_1511 | -0.01138 | 0.526735766 |
| Cyclophosphamide_1512 | -7.70128 | 0.093052302 |
| Pevonedistat_1529 | 0.176286 | 0.883199916 |
| Sapitinib_1549 | -2.76825 | 0.715710949 |
| Uprosertib_1553 | 1.127569 | 0.359992961 |
| LCL161_1557 | 10.47241 | 0.220660898 |
| **Lapatinib_1558** | **-4.08885** | **0.008406023** |
| Luminespib_1559 | -0.01043 | 0.604649115 |
| Alpelisib_1560 | -4.86939 | 0.134386311 |
| Taselisib_1561 | -1.73371 | 0.267621782 |
| EPZ5676_1563 | -3.94869 | 0.416459313 |
| SCH772984_1564 | 0.40252 | 0.933823592 |
| IWP-2_1576 | -0.66744 | 0.197241512 |
| Leflunomide_1578 | -7.56539 | 0.278913463 |
| Entinostat_1593 | 0.152905 | 0.974523006 |
| OSI-027_1594 | 5.809917 | 0.314678599 |
| LGK974_1598 | -3.04781 | 0.416459313 |
| VE-822_1613 | -3.2442 | 0.215826763 |
| WZ4003_1614 | -2.38563 | 0.763971692 |
| CZC24832_1615 | 1.038706 | 0.974523006 |
| AZD5582_1617 | 1.387967 | 0.093052302 |
| GSK2606414_1618 | -2.77108 | 0.20177595 |
| PFI3_1620 | -0.74857 | 0.773739988 |
| PCI-34051_1621 | -0.13713 | 0.696703456 |
| Wnt-C59_1622 | -1.71707 | 0.353283689 |
| I-BET-762_1624 | 1.319432 | 0.510133694 |
| RVX-208_1625 | -3.6064 | 0.578117426 |
| OTX015_1626 | -0.46152 | 0.95415859 |
| GSK343_1627 | -0.33398 | 0.431339592 |
| ML323_1629 | -4.30269 | 0.423861861 |
| Entospletinib_1630 | 0.100315 | 0.696703456 |
| PRT062607_1631 | -1.05443 | 0.706184653 |
| Ribociclib_1632 | 0.623216 | 0.668543435 |
| AGI-6780_1634 | -1.83602 | 0.560734689 |
| Picolinici-acid_1635 | -2.14325 | 0.526735766 |
| AZD5153_1706 | -0.43772 | 0.569394892 |
| CDK9_5576_1708 | -0.07191 | 0.095624165 |
| CDK9_5038_1709 | -0.00586 | 0.340100407 |
| Eg5_9814_1712 | -0.00747 | 0.068222884 |
| ERK_2440_1713 | -0.07117 | 0.706184653 |
| ERK_6604_1714 | 0.517576 | 0.813152501 |
| IRAK4_4710_1716 | -5.46093 | 0.240759873 |
| JAK1_8709_1718 | -2.06291 | 0.267621782 |
| AZD5991_1720 | -8.3396 | 0.290519676 |
| PAK_5339_1730 | -0.40996 | 0.387609425 |
| TAF1_5496_1732 | -4.91942 | 0.093052302 |
| ULK1_4989_1733 | 0.17375 | 0.873124691 |
| VSP34_8731_1734 | -0.7175 | 0.284677193 |
| Selumetinib_1736 | 0.855294 | 0.510133694 |
| IGF1R_3801_1738 | -0.5515 | 0.17566775 |
| JAK_8517_1739 | -2.61211 | 0.066260897 |
| AZD4547_1786 | -1.20171 | 0.340100407 |
| Ibrutinib_1799 | -10.0049 | 0.085663275 |
| Zoledronate_1802 | -2.72891 | 0.100935602 |
| Acetalax_1804 | 0.35751 | 0.883199916 |
| Oxaliplatin_1806 | -15.2192 | 0.284677193 |
| Carmustine_1807 | -39.2129 | 0.050583071 |
| Topotecan_1808 | -0.10322 | 0.578117426 |
| Teniposide_1809 | -0.19849 | 0.314678599 |
| Mitoxantrone_1810 | 0.023645 | 0.773739988 |
| Dactinomycin_1811 | -0.00908 | 0.112249754 |
| Fludarabine_1813 | -3.55844 | 0.493805227 |
| Nelarabine_1814 | -12.9265 | 0.320915725 |
| Fulvestrant_1816 | -7.04599 | 0.115226515 |
| **Vincristine_1818** | **-0.02333** | **0.046092196** |
| Docetaxel_1819 | -0.02523 | 0.076545447 |
| Podophyllotoxin bromide_1825 | -0.02847 | 0.184079493 |
| Dihydrorotenone_1827 | -0.14753 | 0.416459313 |
| Gallibiscoquinazole_1830 | -0.09909 | 0.518400919 |
| Elephantin_1835 | -2.28866 | 0.284677193 |
| Sinularin_1838 | -3.94161 | 0.098251687 |
| Sabutoclax_1849 | -0.0108 | 0.933823592 |
| LY2109761_1852 | -14.0427 | 0.152128355 |
| OF-1_1853 | -1.5667 | 0.883199916 |
| **MN-64_1854** | **-8.00907** | **0.028300664** |
| KRAS (G12C) Inhibitor-12_1855 | -6.53298 | 0.188393753 |
| MG-132_1862 | -0.00205 | 0.813152501 |
| BDP-00009066_1866 | -0.98528 | 0.197241512 |
| Buparlisib_1873 | -0.25304 | 0.109332997 |
| Ulixertinib_1908 | -0.86771 | 0.622629968 |
| Venetoclax_1909 | 0.252282 | 0.86306968 |
| **ABT737_1910** | **-1.77198** | **0.047550955** |
| Dactinomycin_1911 | -0.00062 | 0.438892032 |
| Afuresertib_1912 | -1.00605 | 0.240759873 |
| **AGI-5198_1913** | **-8.55411** | **0.009100075** |
| AZD3759_1915 | -0.58107 | 0.235620097 |
| AZD5363_1916 | -1.32658 | 0.578117426 |
| **AZD6738_1917** | **-1.46405** | **0.046092196** |
| AZD8186_1918 | -2.2171 | 0.302441243 |
| **Osimertinib_1919** | **-0.87047** | **0.014416617** |
| Cediranib_1922 | -0.54363 | 0.273228373 |
| Ipatasertib_1924 | -3.45603 | 0.211068157 |
| GDC0810_1925 | -6.87601 | 0.387609425 |
| GNE-317_1926 | -0.09388 | 0.659255508 |
| GSK2578215A_1927 | -4.01453 | 0.167541583 |
| I-BRD9_1928 | -6.48742 | 0.115226515 |
| Telomerase Inhibitor IX_1930 | -0.17575 | 0.141284189 |
| MIRA-1_1931 | -21.0978 | 0.144831964 |
| **NVP-ADW742_1932** | **-4.01777** | **0.031297274** |
| P22077_1933 | -6.06977 | 0.308520436 |
| Savolitinib_1936 | 0.346235 | 0.803251416 |
| **UMI-77_1939** | **-1.68746** | **0.024686142** |
| WIKI4_1940 | -1.74422 | 0.152128355 |
| Sepantronium bromide_1941 | -0.00032 | 0.974523006 |
| MIM1_1996 | -0.69464 | 0.793381313 |
| **WEHI-539_1997** | **-4.90647** | **0.028300664** |
| BPD-00008900_1998 | -2.26707 | 0.613610946 |
| Foretinib_2040 | -0.23331 | 0.251271278 |
| BIBR-1532_2043 | -8.57319 | 0.09053536 |
| Pyridostatin_2044 | -1.50094 | 0.100935602 |
| AMG-319_2045 | -2.38258 | 0.485745471 |
| MK-8776_2046 | -2.99131 | 0.050583071 |
| Ulixertinib_2047 | -0.49862 | 0.485745471 |
| Vinorelbine_2048 | -0.00128 | 0.366780405 |
| VX-11e_2096 | -0.14784 | 0.92367119 |
| Uprosertib_2106 | 0.50257 | 0.92367119 |
| LJI308_2107 | 2.549836 | 0.994903783 |
| **AZ6102_2109** | **-1.09314** | **0.049047677** |
| GSK591_2110 | 0.662747 | 0.813152501 |
| VE821_2111 | -1.37717 | 0.256643431 |
| AZD6482_2169 | -0.12628 | 0.793381313 |
| AT13148_2170 | 1.019881 | 0.715710949 |
| BMS-754807_2171 | 0.001406 | 0.853036455 |
| JQ1_2172 | 0.021565 | 0.943986599 |

^[1]^ The differences of average IC50 between high eIF3a expressed group and low eIF3a group. P < 0.05 was considered to be significant and highlighted in bold.

**Supplementary Figures**


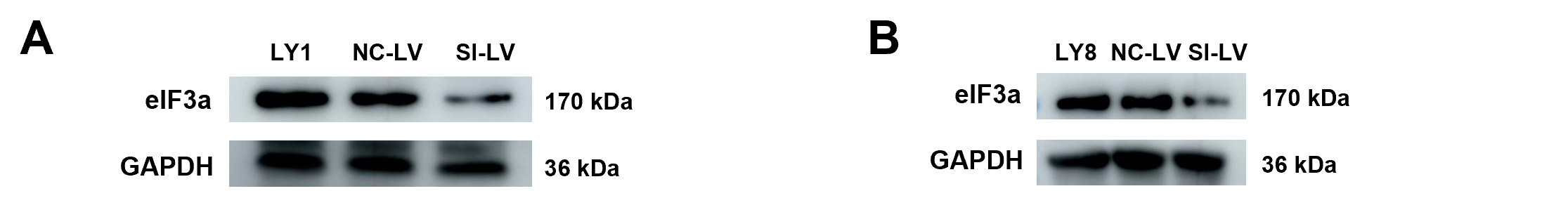


**Figure S1. The knocking results of another siRNA**

(A) DLBCL cells (LY1) transfected with NC-LV and SI-LV. (B) DLBCL cells (LY8) transfected with NC-LV and SI-LV.


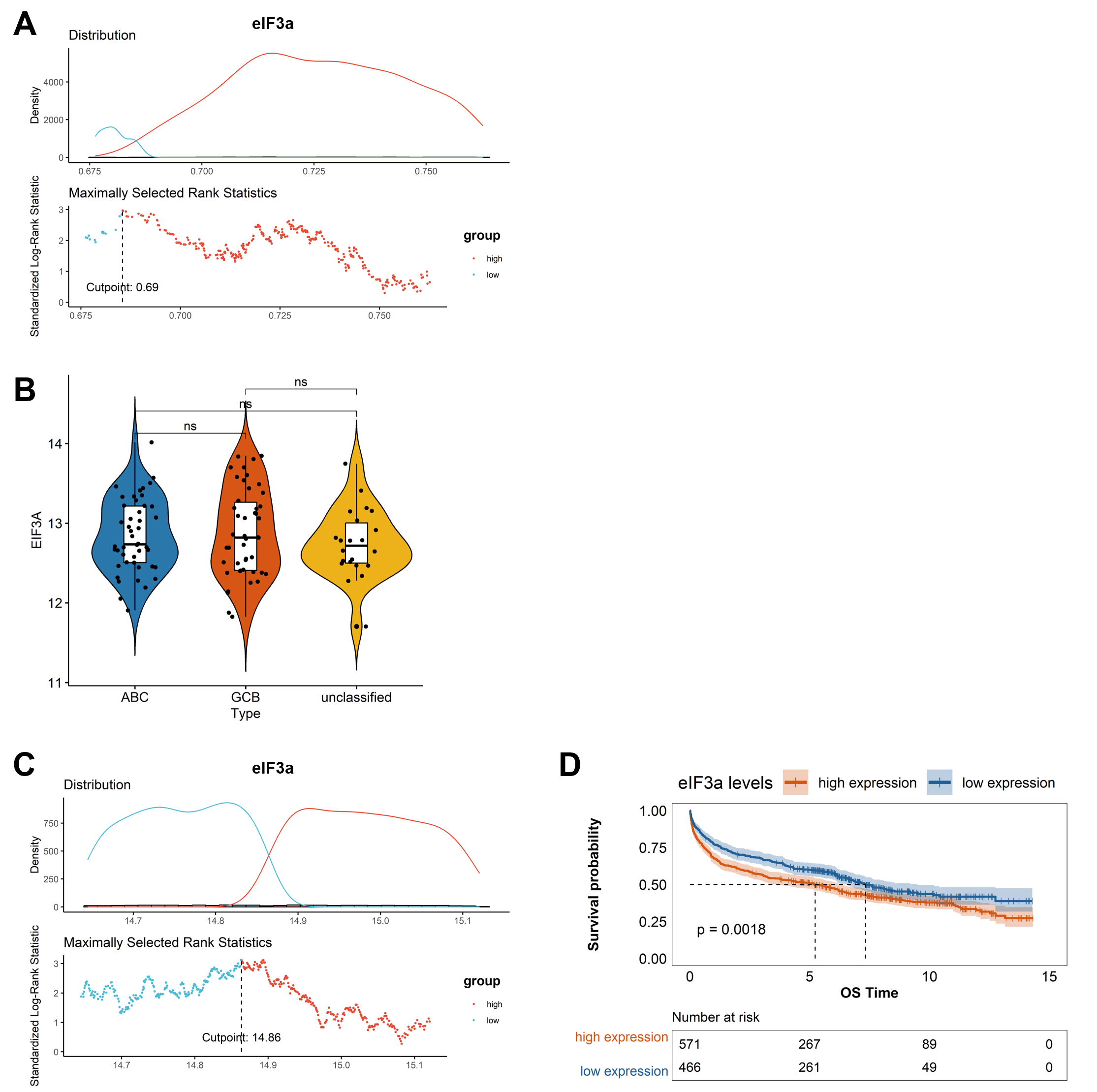


**Figure S2. The prognostic role of eIF3a in several datasets**

(A) The optimum cut value of eIF3a expression in GSE31312. The green line represented “distribution” and red line represented “density”. (B) eIF3a expression in different subtypes of DLBCL in GSE53786. (C) The optimum cut value of eIF3a expression in GSE181063. The green line represented “distribution” and red line represented “density”. (D) Kaplan-Meier curves for OS of DLBCL patients with high- or low- expressed eIF3a levels in GSE181063 (P = 0.0018).
