# Supplementary material for: Eukaryotic initiation factor 3a promotes the development of diffuse large B-cell lymphoma through regulating cell proliferation
Source: BMC Cancer. 2024 Apr 8;24:432. doi: 10.1186/s12885-024-12166-0 (PMC11003032; doi:10.1186/s12885-024-12166-0)
Supplement: Supplementary file 2 — Supplementary Material 2 [file 12885_2024_12166_MOESM2_ESM.docx]

WB original image

Figure 1C


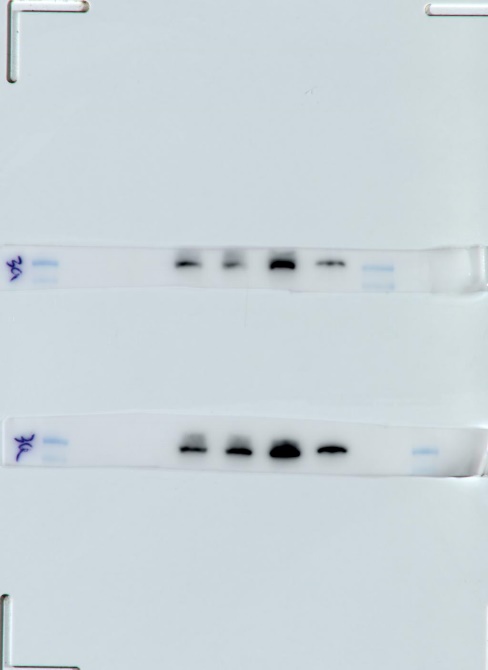


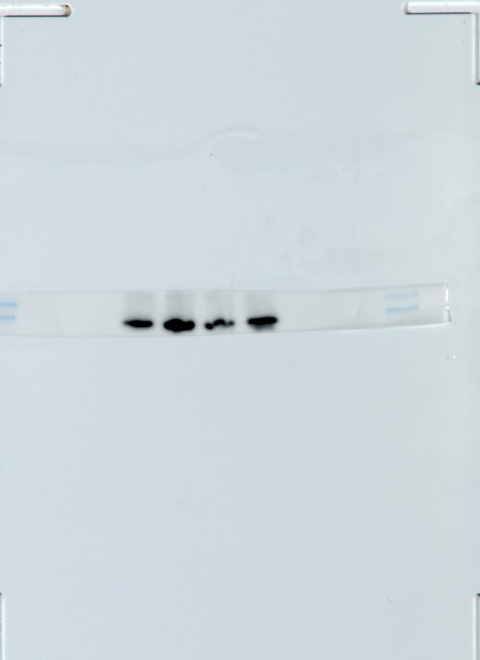


eIF3a

eIF3a

170KDa

170KDa

eIF3a

170KDa

N1 N2 LY1 LY3 LY8 U2932

N1 N2 LY1 LY3 LY8 U2932


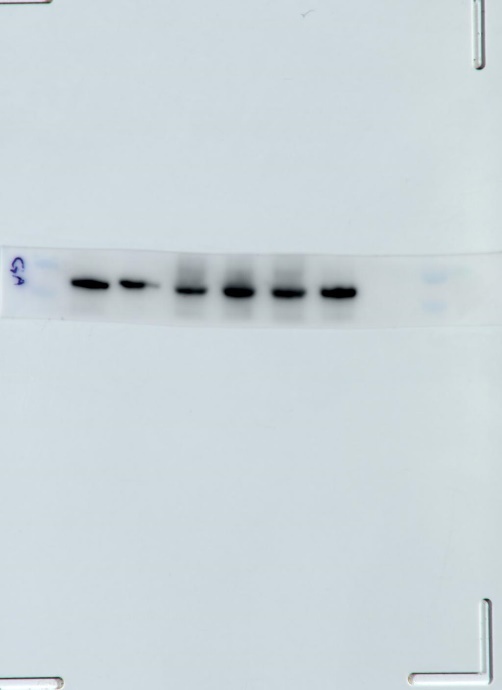

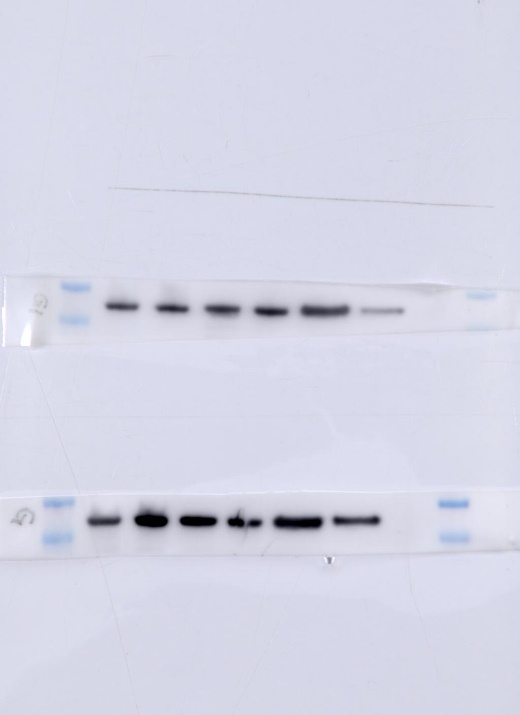


GAPDH

36KDa

36KDa

GAPDH

36KDa

GAPDH

N1 N2 LY1 LY3 LY8 U2932

N1 N2 LY1 LY3 LY8 U2932

Figure 2B


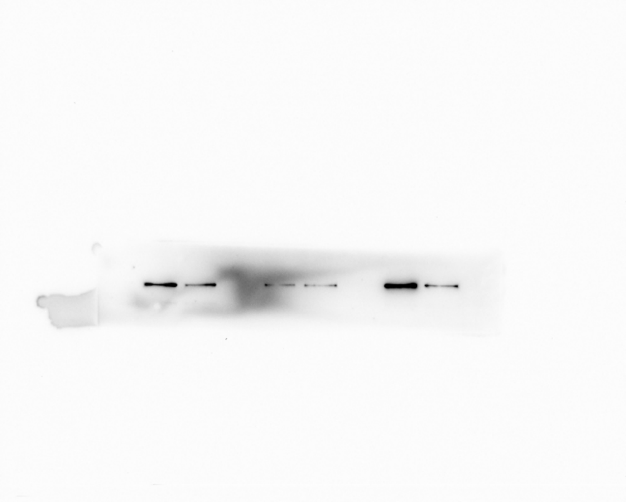

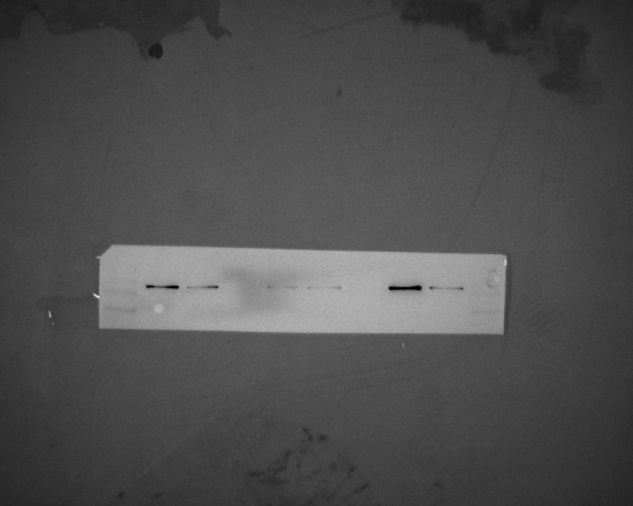


eIF3a

170kDa

NC-LV SI-LV NC-LV SI-LV NC-LV SI-LV

LY1 LY3 LY8

NC-LV SI-LV NC-LV SI-LV NC-LV SI-LV

LY1 LY3 LY8


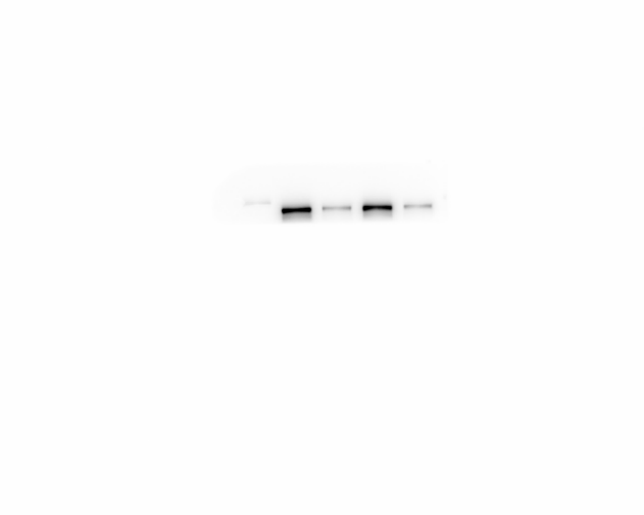

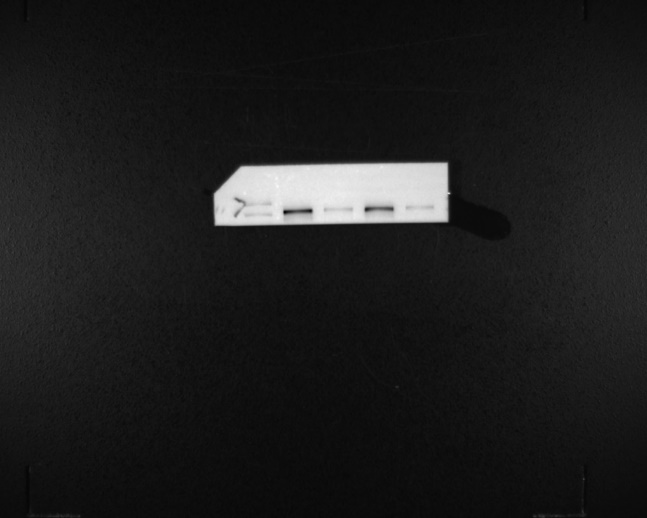


170kDa

eIF3a

NC-LV SI-LV NC-LV SI-LV

LY1 LY8

NC-LV SI-LV NC-LV SI-LV

LY1 LY8


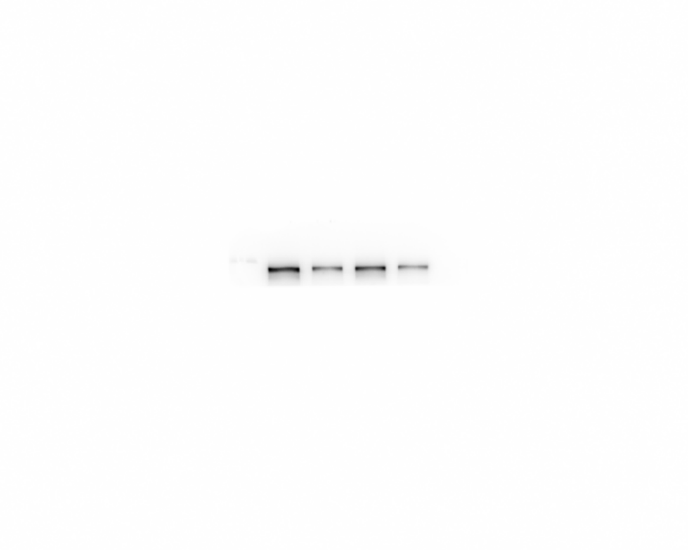

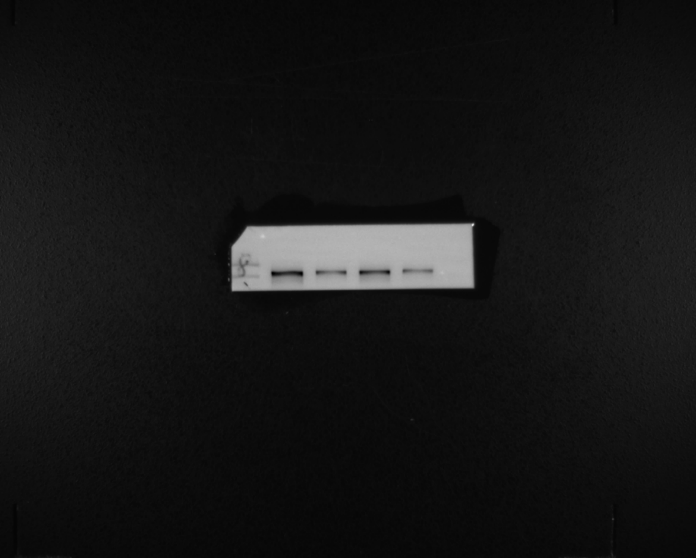


170kDa

eIF3a

NC-LV SI-LV NC-LV SI-LV

LY1 LY8

NC-LV SI-LV NC-LV SI-LV

LY1 LY8


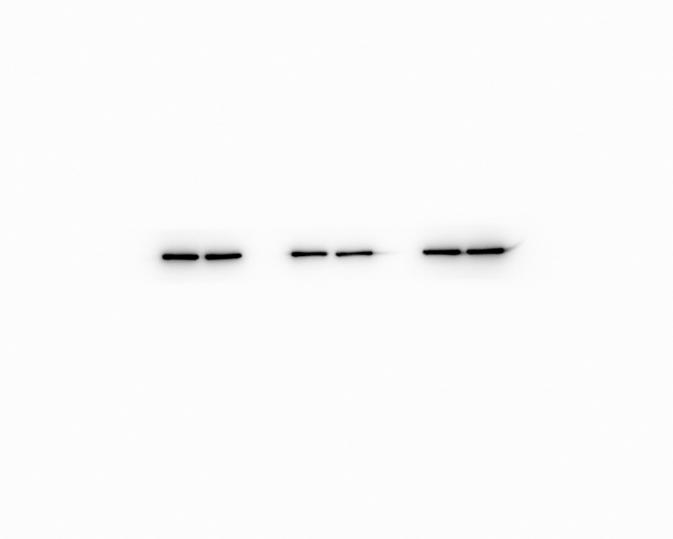

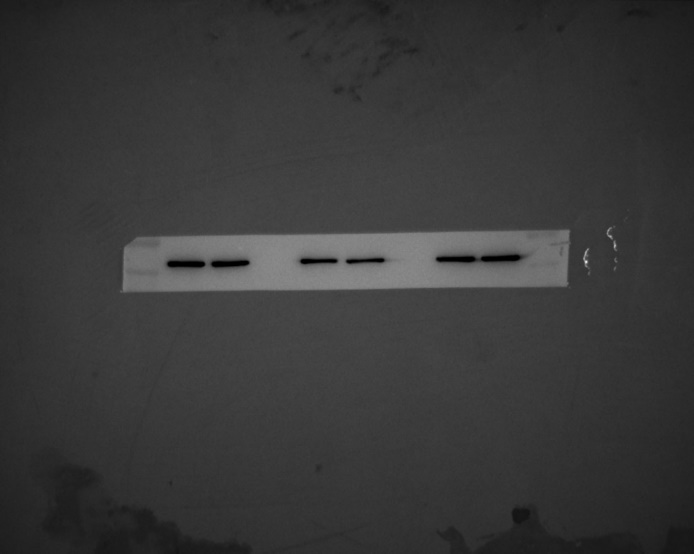


36kDa

36kDa

GAPDH

NC-LV SI-LV NC-LV SI-LV NC-LV SI-LV

LY1 LY3 LY8

NC-LV SI-LV NC-LV SI-LV NC-LV SI-LV

LY1 LY3 LY8


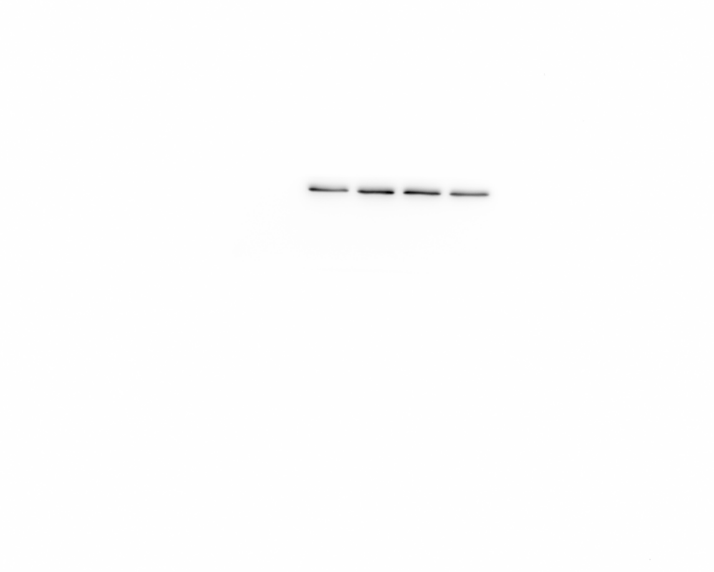

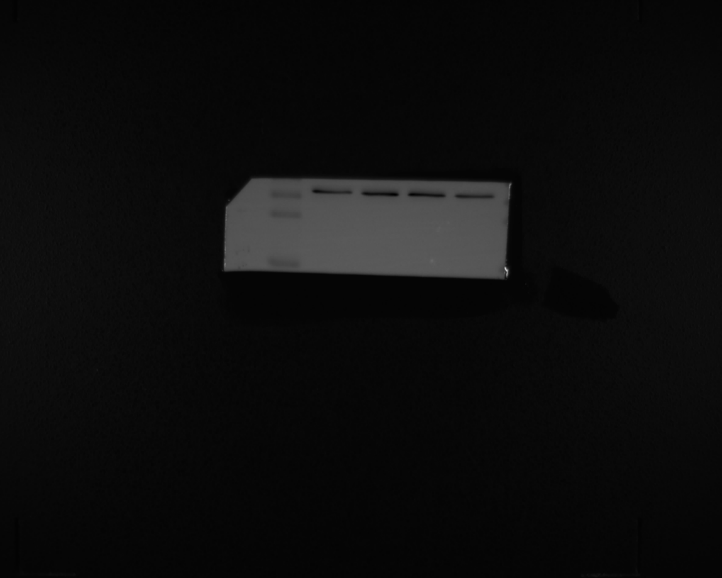


36kDa

GAPDH

NC-LV SI-LV NC-LV SI-LV

LY1 LY8

NC-LV SI-LV NC-LV SI-LV

LY1 LY8


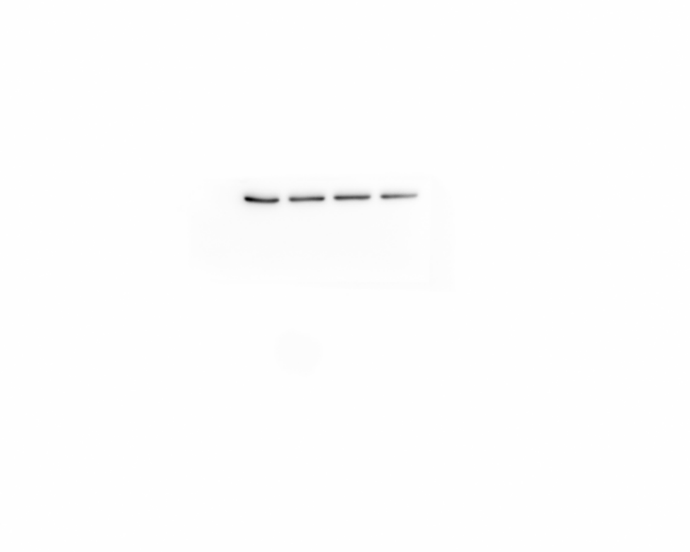

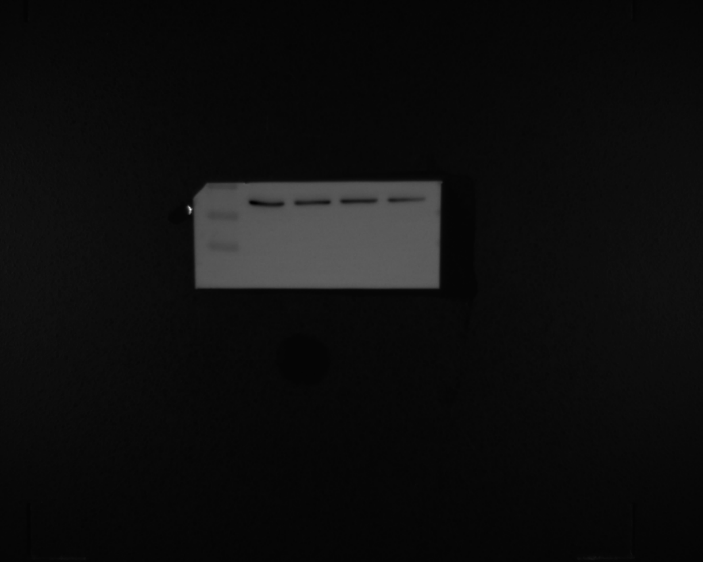


36kDa

GAPDH

NC-LV SI-LV NC-LV SI-LV

LY1 LY8

NC-LV SI-LV NC-LV SI-LV

LY1 LY8

Figure 2E


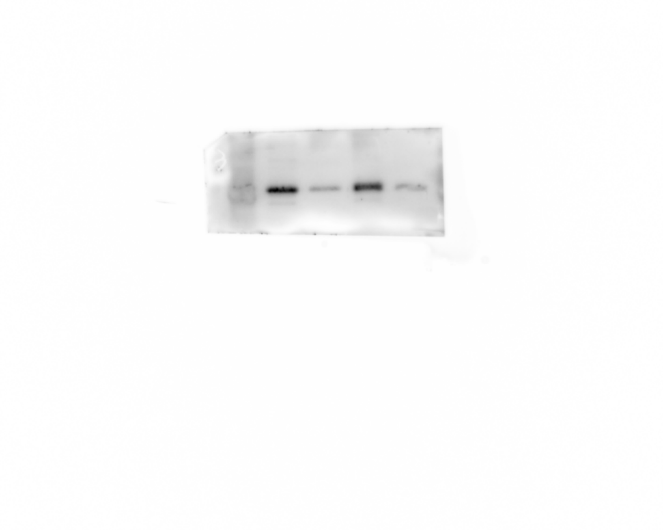

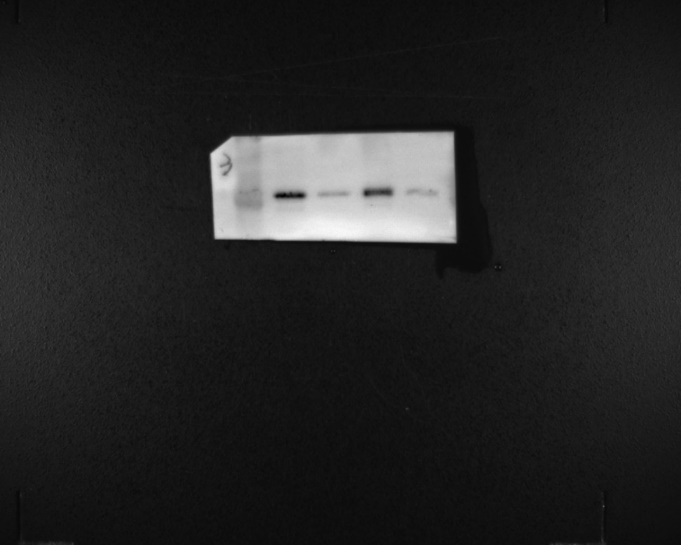


36kDa

Cyclin D1

NC-LV SI-LV NC-LV SI-LV

LY1 LY8

NC-LV SI-LV NC-LV SI-LV

LY1 LY8


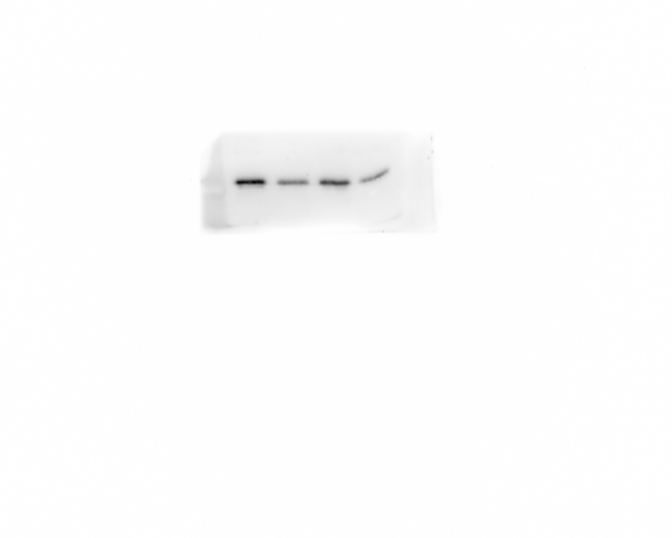

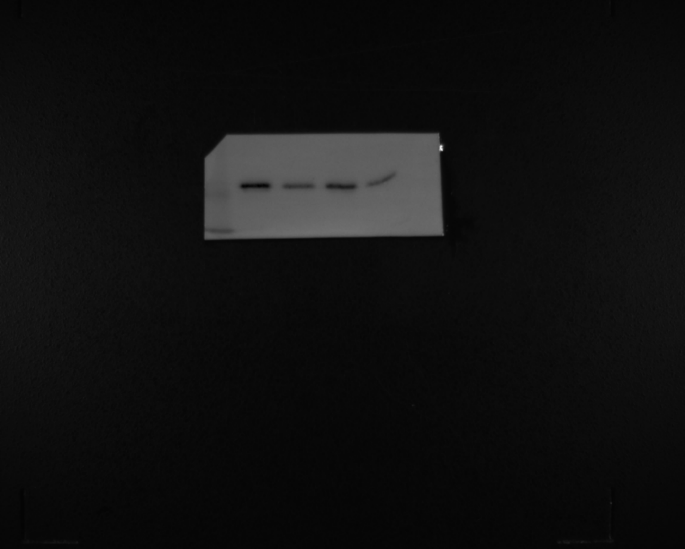


36kDa

Cyclin D1

NC-LV SI-LV NC-LV SI-LV

LY1 LY8

NC-LV SI-LV NC-LV SI-LV

LY1 LY8


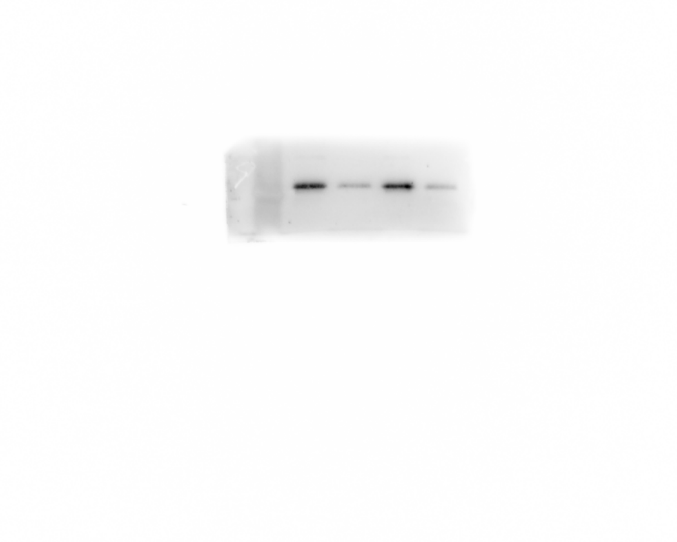

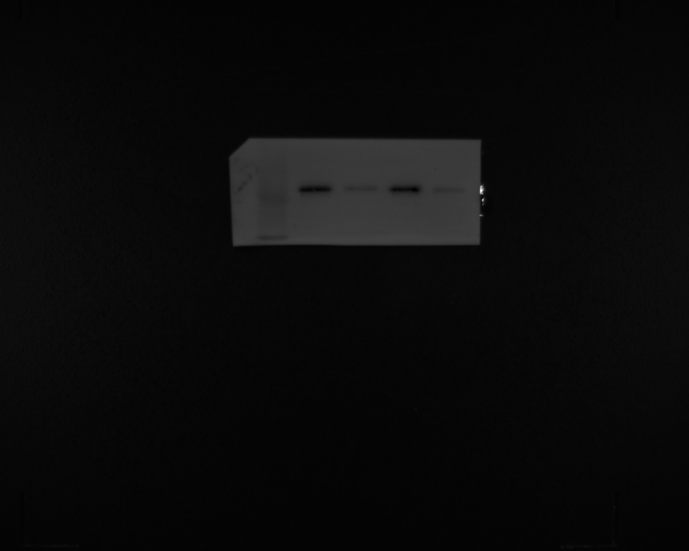


36kDa

Cyclin D1

NC-LV SI-LV NC-LV SI-LV

LY1 LY8

NC-LV SI-LV NC-LV SI-LV

LY1 LY8


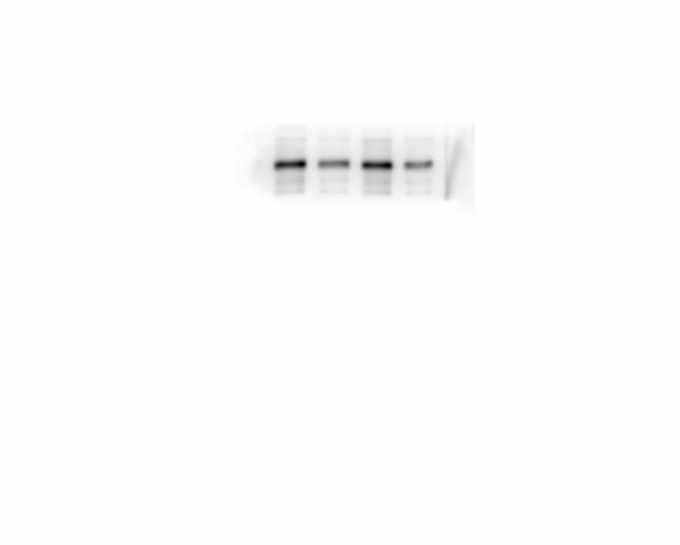

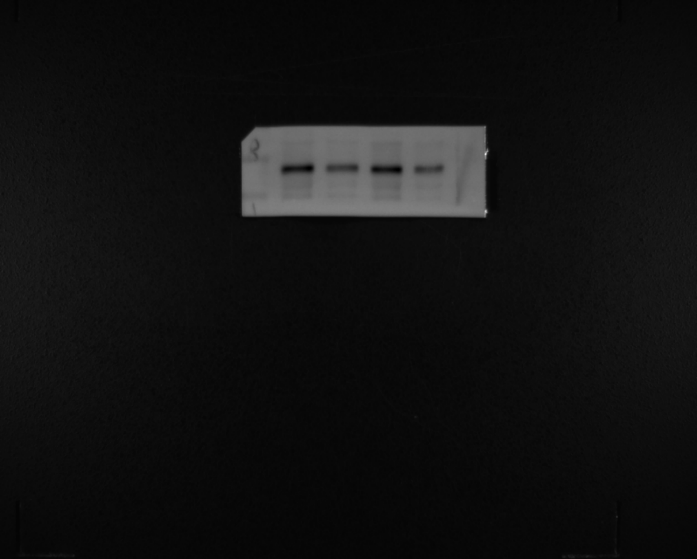


34kDa

CDK4

NC-LV SI-LV NC-LV SI-LV

LY1 LY8

NC-LV SI-LV NC-LV SI-LV

LY1 LY8


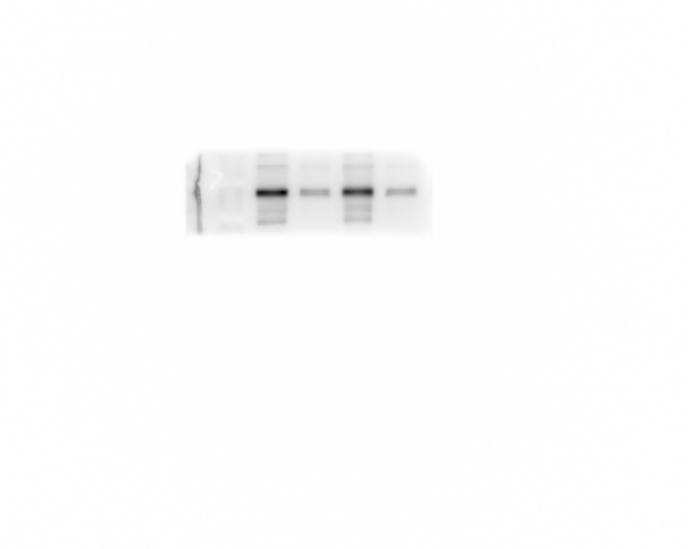

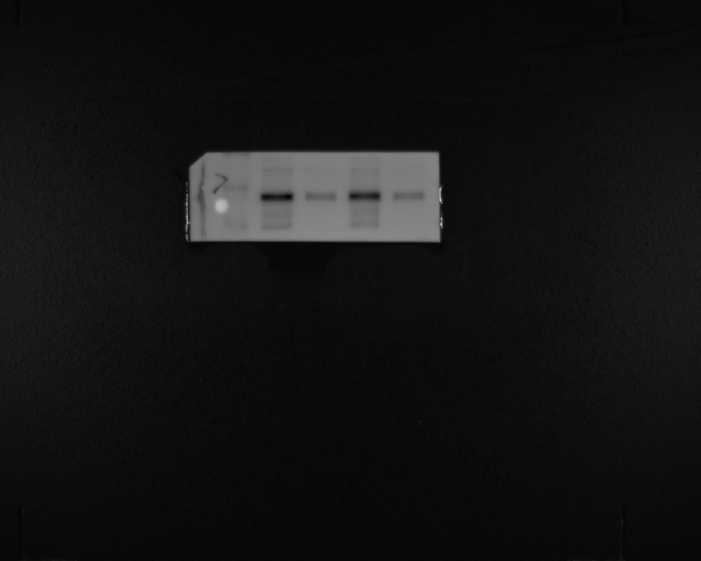


CDK4

34kDa

NC-LV SI-LV NC-LV SI-LV

LY1 LY8

NC-LV SI-LV NC-LV SI-LV

LY1 LY8

NC-LV SI-LV NC-LV SI-LV

LY1 LY8


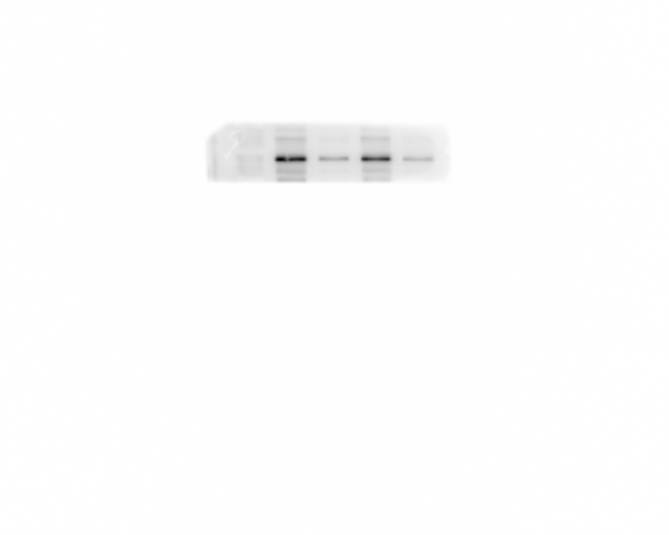

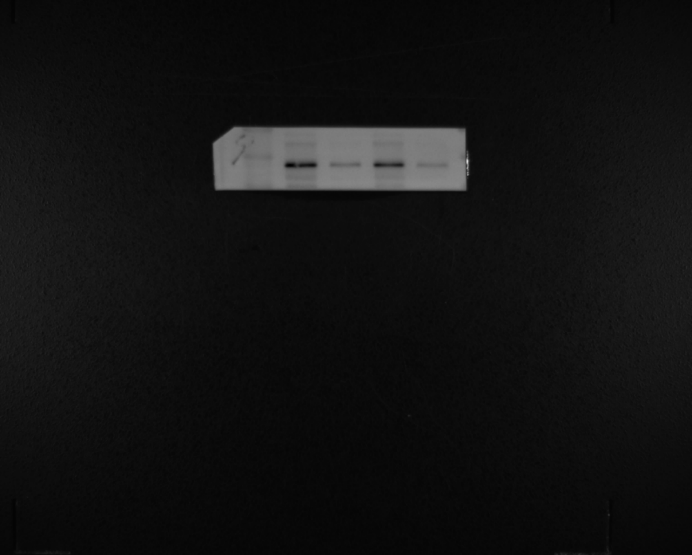


34kDa

CDK4

NC-LV SI-LV NC-LV SI-LV

LY1 LY8

NC-LV SI-LV NC-LV SI-LV

LY1 LY8


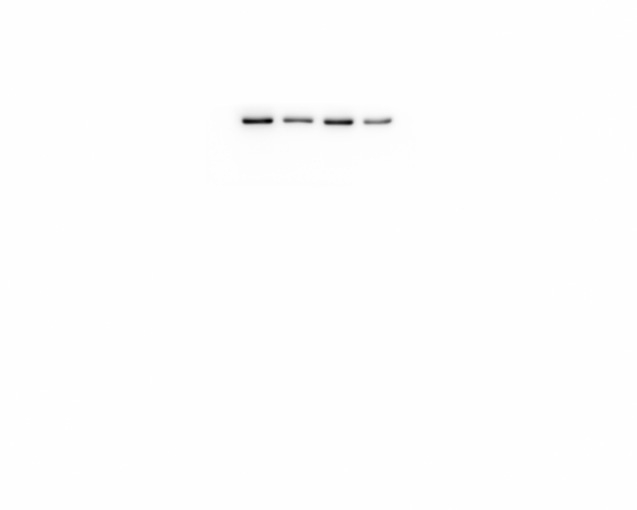

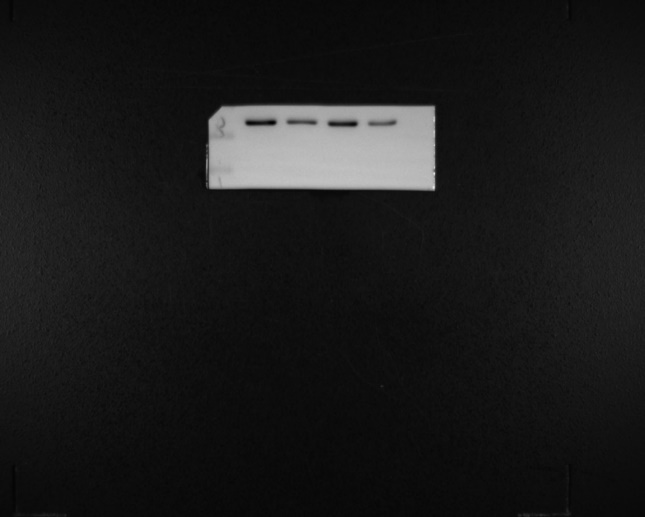


37kDa

CDK6

NC-LV SI-LV NC-LV SI-LV

LY1 LY8

NC-LV SI-LV NC-LV SI-LV

LY1 LY8


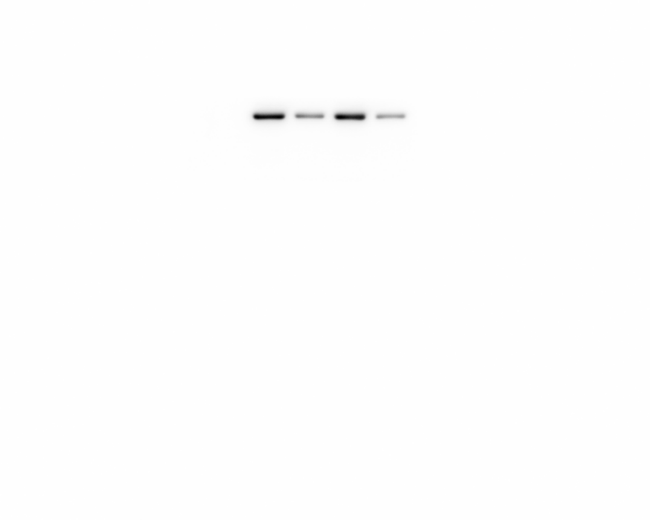

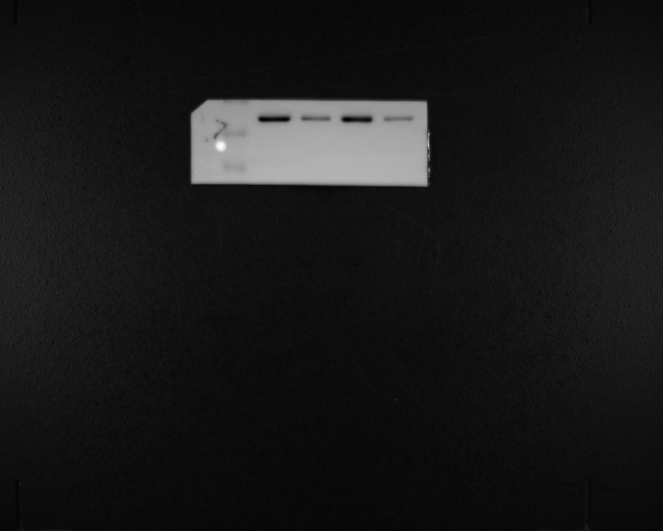


37kDa

CDK6

NC-LV SI-LV NC-LV SI-LV

LY1 LY8

NC-LV SI-LV NC-LV SI-LV

LY1 LY8


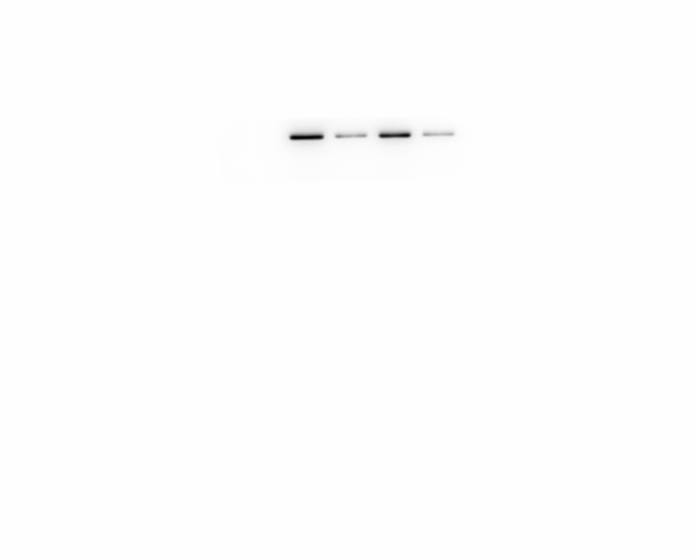

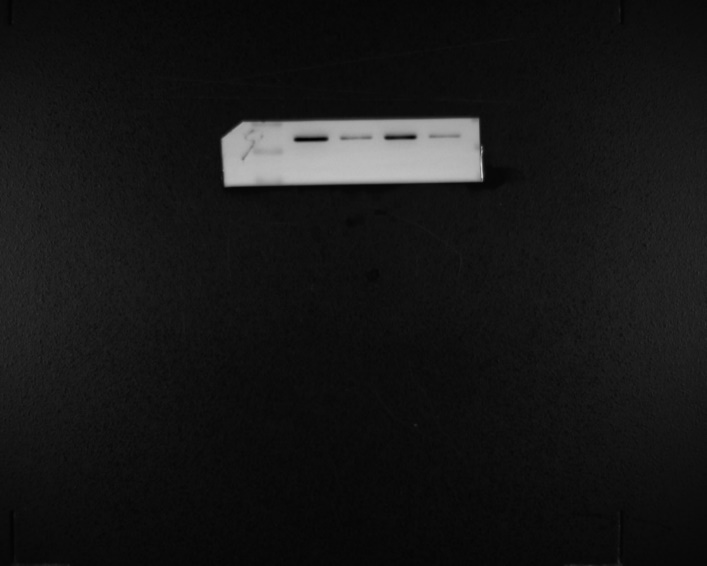


37kDa

CDK6

37kDa

NC-LV SI-LV NC-LV SI-LV

LY1 LY8

NC-LV SI-LV NC-LV SI-LV

LY1 LY8


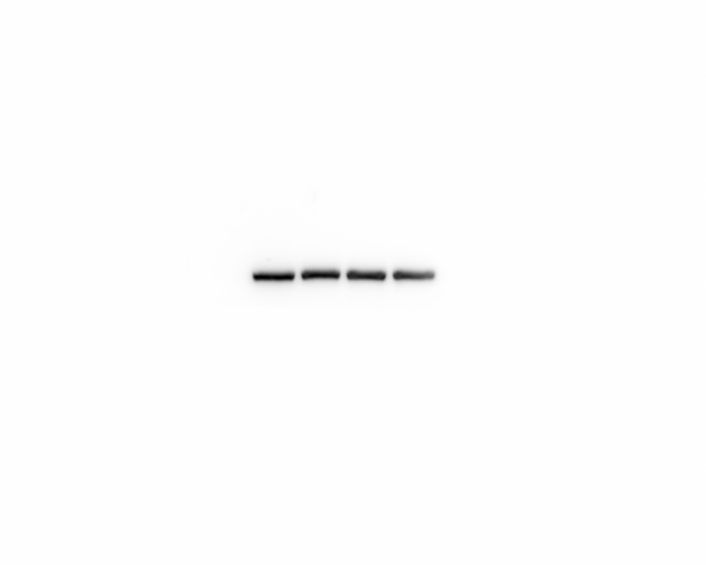

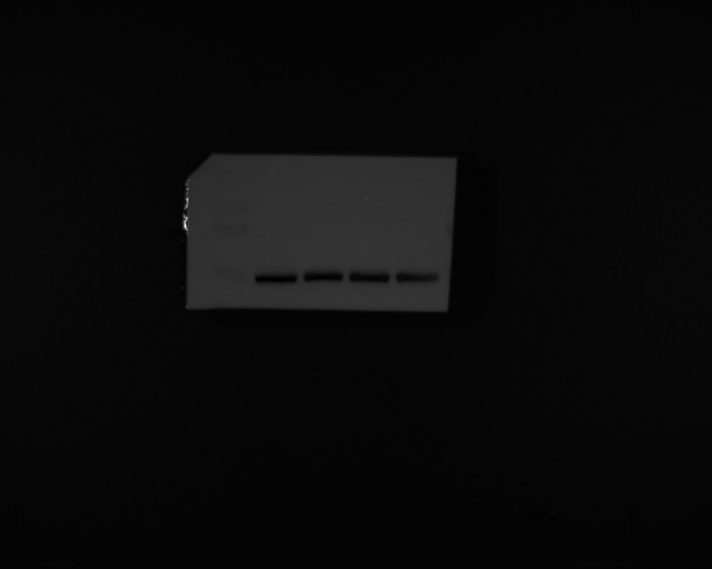


36kDa

GAPDH

NC-LV SI-LV NC-LV SI-LV

LY1 LY8

NC-LV SI-LV NC-LV SI-LV

LY1 LY8


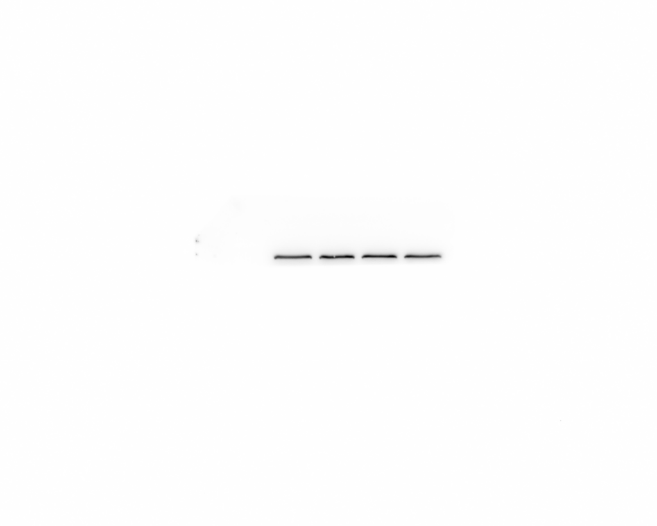

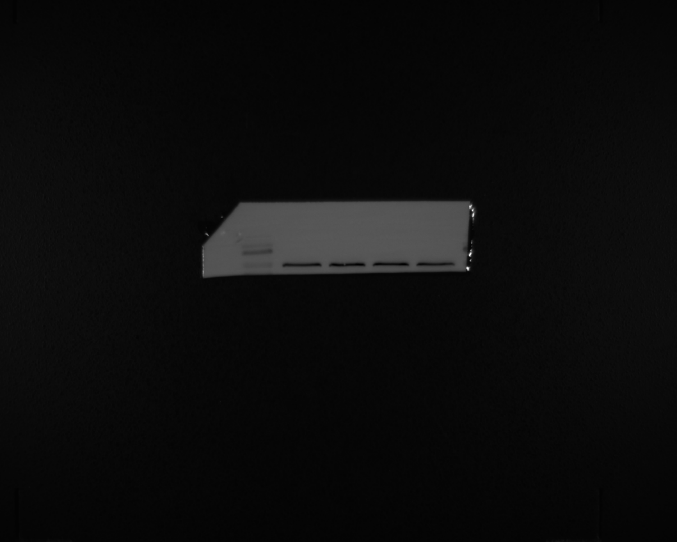


GAPDH

36kDa

NC-LV SI-LV NC-LV SI-LV

LY1 LY8

NC-LV SI-LV NC-LV SI-LV

LY1 LY8


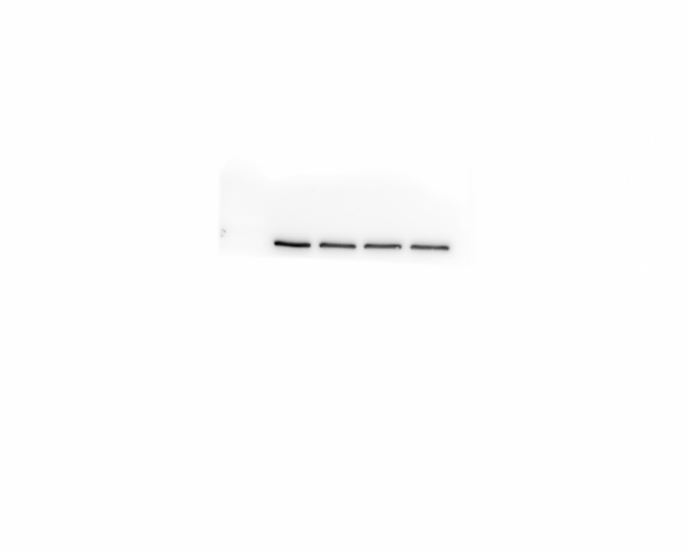

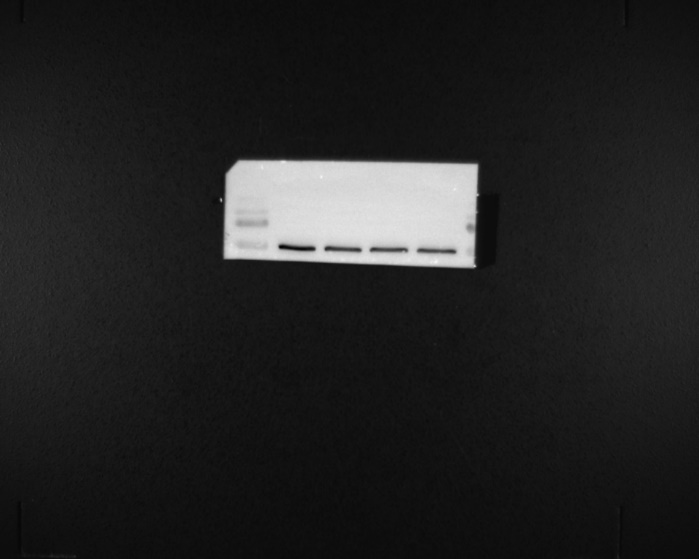


36kDa

GAPDH

NC-LV SI-LV NC-LV SI-LV

LY1 LY8

NC-LV SI-LV NC-LV SI-LV

LY1 LY8

Figure S1


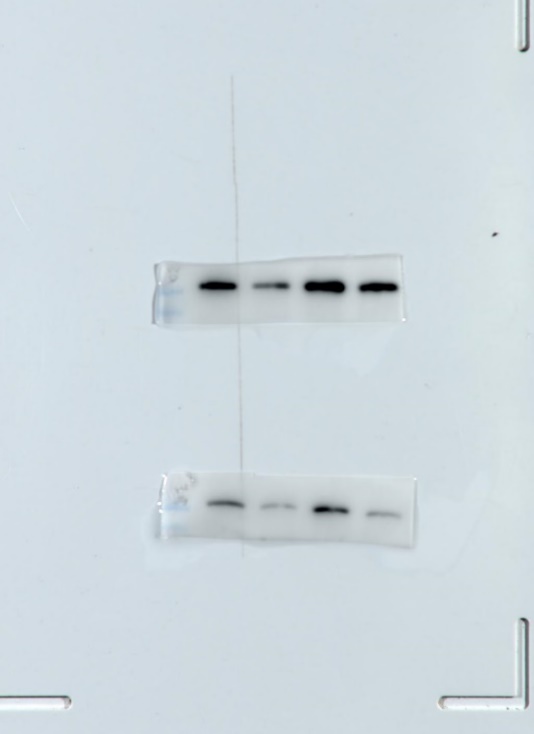

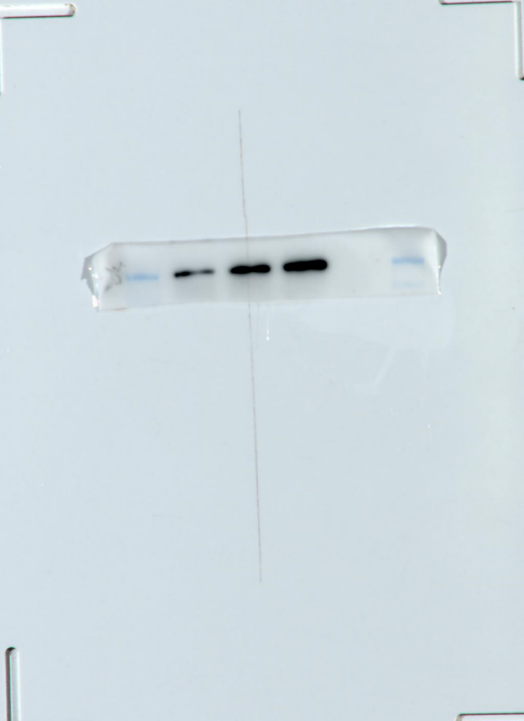


170kDa

eIF3a

170kDa

eIF3a

170kDa

eIF3a

NC-LV SI-LV NC-LV SI-LV

LY1 LY8

SI-LV NC-LV LY1


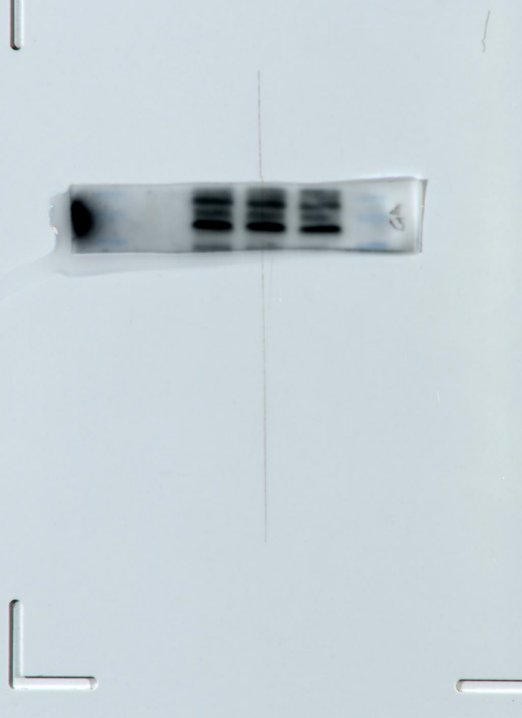

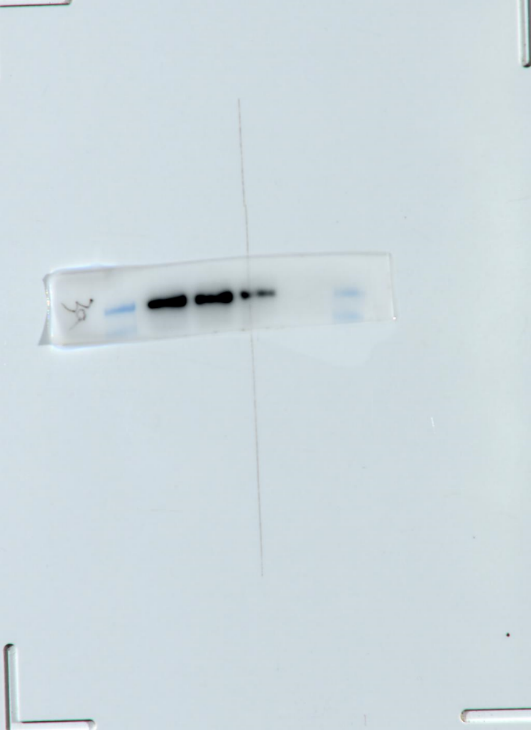


170kDa

eIF3a

36kDa

GAPDH

SI-LV NC-LV LY1

LY8 NC-LV SI-LV


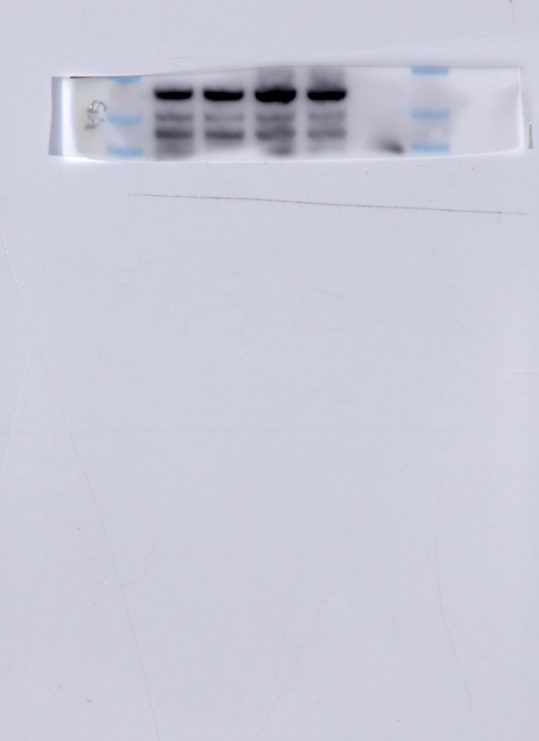

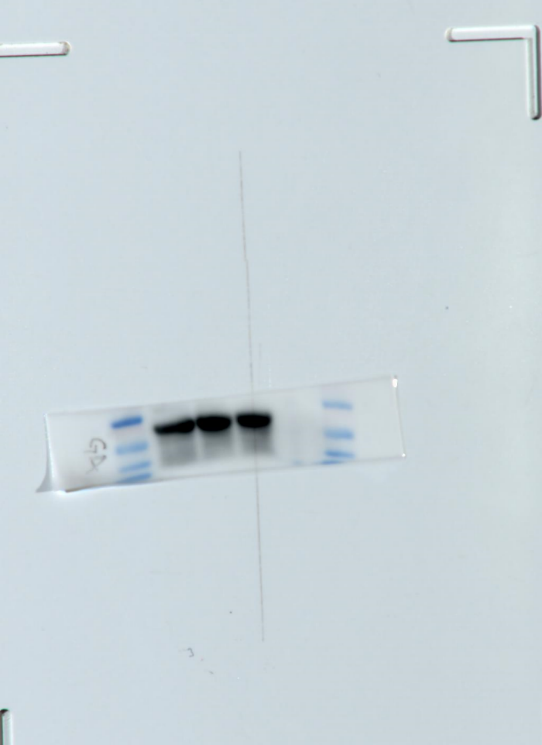


36kDa

GAPDH

36kDa

GAPDH

NC-LV SI-LV NC-LV SI-LV

LY1 LY8

LY8 NC-LV SI-LV


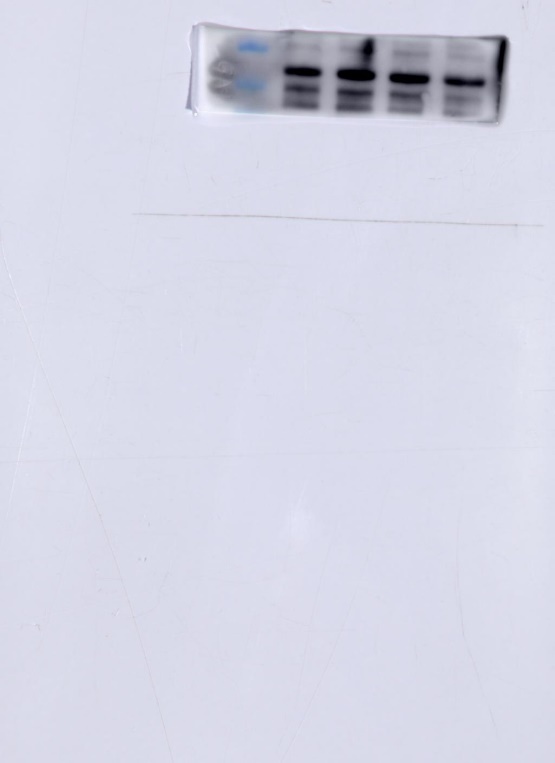


36kDa

GAPDH

NC-LV SI-LV NC-LV SI-LV

LY1 LY8
